# tRNAs Are Stable After All: Pitfalls in Quantification of tRNA from Starved Escherichia coli Cultures Exposed by Validation of RNA Purification Methods

**DOI:** 10.1128/mbio.02805-22

**Published:** 2023-01-04

**Authors:** Thomas Prossliner, Shreya Agrawal, Ditte F. Heidemann, Michael A. Sørensen, Sine L. Svenningsen

**Affiliations:** a Department of Biology, University of Copenhagen, Copenhagen, Denmark; Yale School of Medicine

**Keywords:** *Escherichia coli*, RNA extraction, bacterial stress response, nutrient starvation, rRNA, tRNA

## Abstract

tRNAs and ribosomal RNAs are often considered stable RNAs. In contrast to this view, we recently proposed that tRNAs are degraded during amino acid starvation and drug-induced transcription inhibition. However, reevaluation of our experimental approach revealed that common RNA extraction methods suffer from alarming extraction and size biases that can lead to gross underestimation of RNA levels in starved Escherichia coli populations. Quantification of tRNAs suffers additional biases due to differing fractions of tRNAs with base modifications in growing versus starved bacteria. Applying an improved methodology, we measured tRNA levels after starvation for amino acids, glucose, phosphate, or ammonium and transcription inhibition by rifampicin. We report that tRNA levels remain largely unaffected in all tested conditions, including several days of starvation. This confirms that tRNAs are remarkably stable RNAs and serves as a cautionary tale about quantification of RNA from cells cultured outside the steady-state growth regime. rRNA, conversely, is extensively degraded during starvation. Thus, E. coli downregulates the translation machinery in response to starvation by reducing the ribosome pool through rRNA degradation, while a high concentration of tRNAs available to supply amino acids to the remaining ribosomes is maintained.

## INTRODUCTION

The Escherichia coli responses to nutrient starvation are manifold, involving redirected enzyme activities and physiological changes such as reduced cell size, altered membrane composition, and increased thickness of the peptidoglycan layer (1). The major changes in gene expression that govern these responses include strongly reduced synthesis of rRNA and tRNA. These noncoding RNAs are often collectively referred to as “stable RNA” due to their greatly increased half-lives compared to short-lived mRNA. However, it has become clear that these RNAs are not universally stable but, instead, can be degraded under certain unfavorable growth conditions. Multiple studies in Escherichia coli have shown that nutrient starvation and entry into the stationary phase can trigger pathways that lead to fragmentation and eventual degradation of rRNAs (2–7). Similarly, mechanisms are in place to dispose of misprocessed rRNAs and tRNAs during exponential growth (2, 6, 8, 9). In agreement with the notion that components of the bacterial translation machinery are degraded when the cell encounters unfavorable conditions, our group published data suggesting that tRNAs are highly unstable during short-term amino acid starvation in E. coli (10–12). Based on our data, we concluded that tRNAs are indiscriminately and rapidly degraded during starvation. However, despite intensive efforts, we were unable to identify the enzyme(s) responsible for the apparent degradation of tRNAs (10, 12). In addition, although the results obtained in the study were highly reproducible using the same approach, continued research carried out in our laboratory produced contradictory results when alternative protocols were used. In particular, applying different RNA extraction methods yielded results inconsistent with our previous findings. To investigate the reason behind these inconsistencies we undertook an in-depth reassessment of tRNA stability during stress conditions: first, we evaluated our quantification approach, in particular, isolation and detection of RNA species, and devised an improved method; second, we applied this improved method to reassess the dynamics of tRNA degradation during early amino acid starvation and rifampicin-induced transcription inhibition; and third, we extended our analysis to short- and long-term starvation for a variety of nutrients.

Here, we establish that tRNAs are indeed remarkably stable, even compared to other stable RNAs such as rRNA. We reveal severe drawbacks of commonly used RNA extraction methods that led to our previous erroneous conclusion and, importantly, could affect the quantification of any RNA in similar conditions, independently of the type of downstream analysis. Our findings underscore the need for thorough evaluation of methodology when setting out to quantify any RNA species from bacteria that are cultured outside the steady-state growth regime, and we hope that the work presented here can serve as an example and perhaps a guideline to avoid similar misinterpretations of future experiments.

## RESULTS

### The growth state of E. coli can affect the efficiency of common RNA extraction methods.

**FIG 1:**
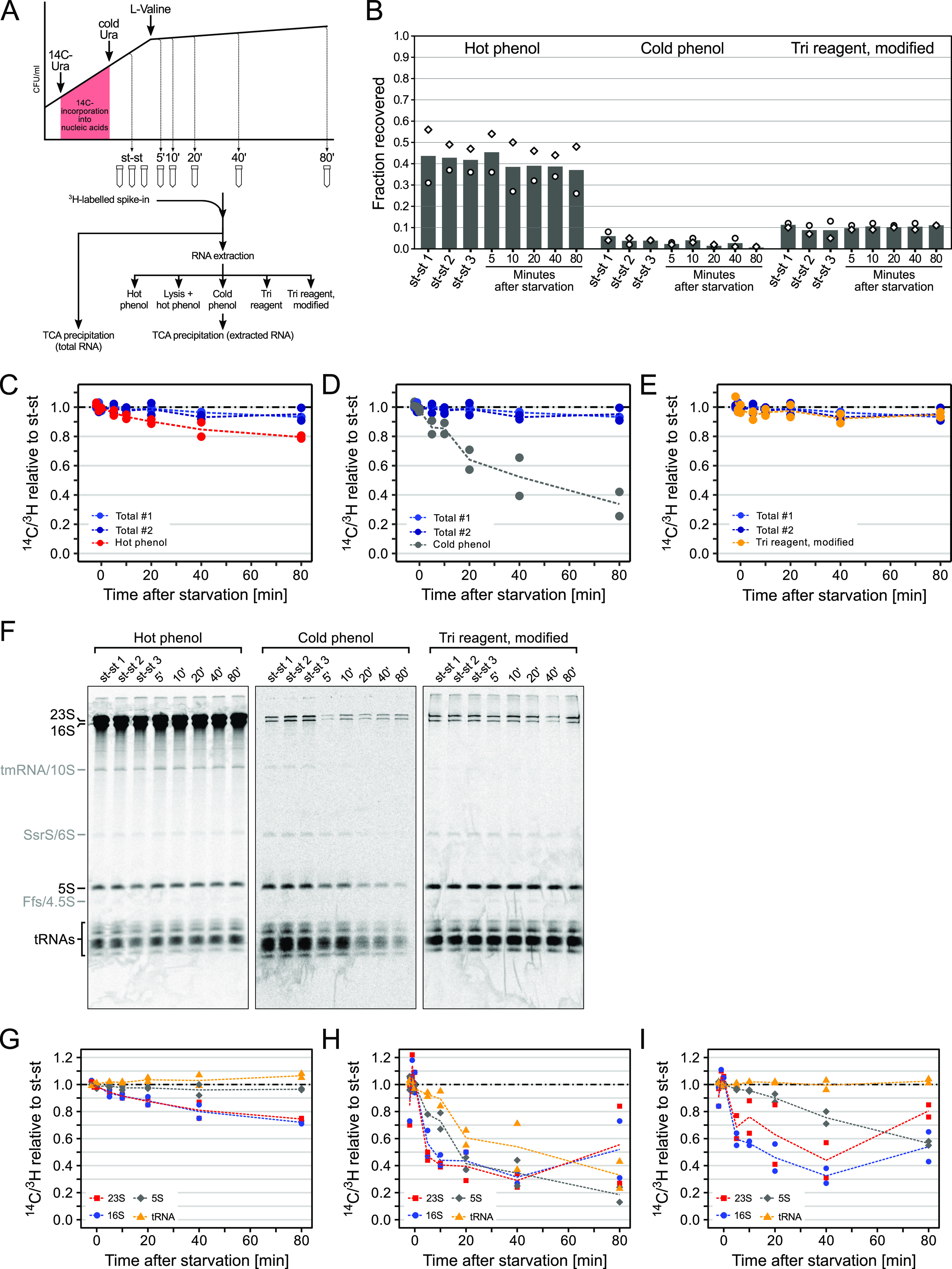
Growth state-dependent efficiency and size bias of common RNA extraction methods. (A) Schematic representation of RNA extraction efficiency measurement by [^14^C]-radiolabeling. Cells were grown in MOPS minimal medium supplemented with [^14^C]-uracil for approximately two generations before adding nonradioactive uracil. Subsequently, samples were taken during steady-state growth and during valine-induced isoleucine starvation. Samples were spiked in by addition of a small volume of [^3^H]-labeled cell culture harvested during steady-state growth and either directly subjected to TCA precipitation as an estimate for total RNA or extracted by a selection of common RNA extraction methods. Extracted RNA was analyzed further or precipitated and compared to total, TCA-precipitable RNA to estimate overall extraction efficiency. (B) Overall efficiency of common RNA extraction methods. The efficiency of hot phenol, cold phenol, and modified TRI Reagent extractions was determined as the fraction of recovered RNA out of total RNA. Circles indicate data points of two independent biological replicates; bars indicate the average of the two measurements. Shown is the RNA recovery in three samples obtained during steady-state growth (st-st 1 to 3) and 5, 10, 20, 40, and 80 min after induction of isoleucine starvation. (C to E) Efficiency of RNA extraction from starved cultures relative to steady-state cultures. The relative efficiency of hot phenol (C), cold phenol (D), and modified TRI Reagent (E) extractions was determined as the fraction of recovered RNA after starvation relative to the mean of three steady-state samples. For comparison, the fraction of total, TCA-precipitable RNA relative to the steady state is indicated in blue (dark and light blue for two independent biological replicates). Circles represent data points of two independent biological replicates; dotted lines represent the average of the two measurements. (F) PAGE analysis of radiolabeled RNA obtained by hot phenol, cold phenol, and modified TRI extraction. [^14^C]-labeled RNA was separated on denaturing 5% polyacrylamide gels and detected by phosphor imaging. Bands corresponding to major RNA species (rRNAs and tRNAs) are indicated in black. Bands attributed to other abundant RNA species (tmRNA/10S RNA, SsrS/6S RNA, and Ffs/4.5S RNA) are indicated in gray. (G to I) Efficiency of extraction of individual RNA species by hot phenol (G), cold phenol (H) and modified TRI Reagent (I) extraction from starved cultures relative to steady-state cultures. RNA bands detected in panel F corresponding to 23S, 16S, and 5S rRNA and tRNAs were cut out and RNase-treated, and the incorporated radioactivity was measured to obtain the [^14^C]/[^3^H] ratio for normalization. Filled symbols represent data points of two independent biological replicates; dotted lines represent the average of the two measurements. The dash-dotted line indicates the steady-state level.

In order to accurately quantitate the relative level of tRNAs (and any RNA) in changing growth conditions, it is crucial that RNA extraction fulfils a number of criteria. First, it must yield total RNA of sufficient quantity and quality for the chosen downstream analysis. Second, the efficiency of RNA extraction must be independent of physiological changes to the cells that occur in response to the change of growth conditions. And third, the relative extraction efficiency of individual RNA species from the same sample must be independent of the state of the culture. To test if RNA extraction methods fulfil these criteria, we conducted a thorough assessment of three commonly used extraction protocols (Fig. 1A, schematic). In brief, we radiolabeled cellular RNA and DNA from exponentially growing cells of wild-type E. coli K-12 MAS1081 (MG1655 *rph*^+^ *gatC*^+^ *glpR*^+^) using [^14^C]-uracil. Total RNA was extracted from samples harvested before and after isoleucine starvation. For normalization, we spiked all [^14^C]-labeled cell samples with a small volume of unstarved cells cultured in the presence of [^3^H]-uridine before extraction. The tested extraction methods were (i) cold phenol extraction, which was originally developed as a mild extraction procedure that maintains the aminoacyl charge on tRNAs (12, 13), (ii) hot phenol extraction (3, 14), which is known to give a high yield of RNA, performed with and without an initial step of cell lysis by sonication, and (iii) the commercially available TRI Reagent extraction method, a convenient and rapid method for RNA extraction, carried out with and without additional vortexing of the samples (12).

First, we determined the overall yield of RNA extraction by determining the fraction of [^14^C]-uracil in extracted RNA relative to the total incorporated, trichloroacetic acid (TCA)-precipitable [^14^C]-uracil in RNA from the cultures. The [^14^C]-TCA-precipitable material is a measure of pyrimidines found in RNA and DNA polymers longer than ~16 nucleotides and can therefore be used as an estimate of total RNA when the alkali-stable DNA component is subtracted (15), which we did for all values presented in Fig. 1. As shown in Fig. 1B, hot phenol extraction recovered between 31 and 56% of the incorporated label in the three samples from balanced growth conditions (steady-state [st-st] 1 to 3). After induction of isoleucine starvation, the relative yield remained at a similar level throughout the measured time frame. Addition of a sonication step to increase cell lysis preceding hot phenol extraction did not improve the yield (see Fig. S1A in the supplemental material). In contrast, the relative yield of RNA from cold phenol extraction was consistently below 10% and decreased during starvation relative to cultures in balanced growth. Extraction with TRI Reagent yielded an efficiency of approximately 10% from both starved and unstarved samples. Following the manufacturer’s protocol (i.e., omitting vortexing steps during extraction) resulted in a slight overall decrease of yield compared to the modified protocol previously used in our laboratory (Fig. S1A).

10.1128/mbio.02805-22.1 FIG S1 Modification of hot phenol and TRI Reagent extraction protocols does not affect extraction. Download FIG S1, PDF file, 0.7 MB. Copyright © 2023 Prossliner et al. 2023 Prossliner et al. https://creativecommons.org/licenses/by/4.0/ This content is distributed under the terms of the Creative Commons Attribution 4.0 International license.

These results highlight the variable RNA yield across commonly applied RNA extraction methods, and point toward a negative bias of RNA extraction from starved cells. Therefore, we determined the change in overall RNA extraction efficiency after starvation by determining the [^14^C]/[^3^H] ratio in starvation samples relative to samples harvested from the same culture in balanced growth. We observed a decrease of the [^14^C]/[^3^H] ratio in total, TCA-precipitable RNA to approximately 95% after 80 min of starvation (Fig. 1C to E). When RNA was extracted by hot phenol, we saw a steadily declining [^14^C]/[^3^H] ratio to approximately 80% of steady-state levels after 80 min of starvation (Fig. 1C). This decrease of total RNA extracted from starved cells is consistent with previous observations that rRNA, which constitutes the majority of total cellular RNA, is partially degraded after isoleucine starvation (3). RNA degradation can also explain the slight decrease of the [^14^C]/[^3^H] ratio in total, TCA-precipitable material, as it results in an increase of mono-, di- and short oligomers that are not captured in the TCA precipitate (16). Additional lysis by sonication prior to hot phenol extraction yielded a similar result, although we observed a more rapid drop in the initial 10 min of starvation (Fig. S1B). Conspicuously, cold phenol extraction, which was the main method used in our previous study of tRNA stability (12), showed a steep decrease of the [^14^C]/[^3^H] ratio to approximately 35% after 80 min of starvation (Fig. 1D). In contrast, both TRI Reagent protocols maintained an almost constant [^14^C]/[^3^H] ratio close to the ratio in total TCA-precipitable RNA throughout the experiment (Fig. 1E; Fig. S1C). Taken together, these results show that amino acid starvation can have a considerable effect on the RNA extraction efficiency, and this is dependent on the method chosen for RNA extraction.

### RNA extraction methods can exhibit both inherent and growth-state-dependent size biases.

Having established the differences in efficiency of total RNA extraction, we next investigated the potential bias of the extraction methods with regard to RNA size by separating radiolabeled RNA using denaturing polyacrylamide gel electrophoresis (PAGE), analyzing the overall size distribution, and quantifying changes in extraction efficiency of selected RNA species. As shown in Fig. 1F and Fig. S1D, all extraction methods yielded visible bands corresponding to ribosomal RNAs (23S, 16S, and 5S) and tRNAs. In addition, several fainter bands could be detected, which we attribute to transfer-messenger RNA (tmRNA/10S RNA), the RNA polymerase-associated 6S RNA, and the RNA component of the signal recognition particle, 4.5S RNA. Although all of these RNAs could be detected in all RNA preparations, PAGE analysis revealed strikingly different patterns dependent on the RNA extraction protocol. While hot phenol extraction yielded the expected pattern throughout the sampling time, with the majority of RNA consisting of 23S and 16S rRNA, both cold phenol and TRI Reagent extraction showed a clear size bias and contained mostly tRNA and 5S rRNA. In addition, RNA prepared with cold phenol displayed an overall reduction in the intensity of all visible bands after starvation.

To be able to quantitate the isotope ratio ([^14^C]/[^3^H]) of distinct bands on the PAGE gels, we cut out the bands of interest for analysis in a scintillation counter (see Materials and Methods). The tRNA bands were analyzed as a whole, and in samples prepared by hot phenol extraction, the tRNA fraction showed an increase to nearly 110% after 80 min of starvation (Fig. 1G; Fig. S1E). Importantly, in samples prepared by cold phenol, the tRNA fraction was reduced to approximately 40% of the steady state level after 80 min of starvation (Fig. 1H), while it did not show a change in abundance for samples prepared with TRI Reagent or the modified TRI Reagent protocol (Fig. 1I; Fig. S1F). This discrepancy between the methods clearly demonstrated that the decrease in cold phenol-extracted tRNA levels upon starvation was largely a result of the extraction method, and not, as we had erroneously concluded, due to starvation-induced tRNA degradation.

For the bands corresponding to rRNA in samples extracted by hot phenol, we observed a decrease of 23S and 16S rRNA to approximately 75% and a decrease of 5S rRNA to approximately 95% of the steady-state level (Fig. 1G). Samples that were sonicated prior to RNA extraction yielded comparable results, although 23S and 16S rRNA were reduced to a minor extent (Fig. S1E). Reductions in rRNA are consistent with previous measurements of rRNA degradation during isoleucine starvation (3). In the previous study, the RNA was extracted by hot phenol, and rRNA degradation was confirmed by methods that do not require RNA extraction, namely, by fluorescence *in situ* hybridization of 16S rRNA, and an increase in formic acid-soluble RNA degradation products upon starvation was observed (3). rRNA extracted by cold phenol showed decreases in 23S, 16S, and 5S rRNA to approximately 40% after 80 min (Fig. 1H). In samples obtained using the modified TRI Reagent protocol, 23S and 16S rRNAs showed a surprising trend of first decreasing upon starvation before increasing again at later time points. 5S rRNA declined monotonically to approximately 60% after 80 min (Fig. 1I). This trend was similar when using the conventional TRI reagent protocol, although the rise in 23S and 16S rRNA in the last 40 min was not observed (Fig. S1F).

Taken together, these results demonstrate that both cold phenol and TRI Reagent extractions are inherently biased against large RNAs, including rRNAs. In addition, the efficiency of recovery of all tested RNA species by cold phenol extraction appears to be highly influenced by the growth state of the cell and decreases to a fraction of the steady-state efficiency after starvation. TRI Reagent extraction shows a similar bias that increases in severity with the size of the RNA: tRNAs are extracted with consistent efficiency, while larger RNAs are extracted poorly overall and to different extents from growing and starved cells. The hot phenol protocol recovered the major cellular RNAs in the expected relative proportions and did not show a growth-state-dependent bias in extraction efficiency. We conclude that hot phenol extraction is a suitable protocol for quantitative extraction of RNA from both growing and nutrient-starved E. coli.

The cold phenol method was developed to preserve tRNA charging levels during extraction (13). We compared the charging levels of four abundant tRNAs in RNA samples obtained by hot and cold phenol extraction and found that the purification method did not affect the charging levels (Fig. S2). This result suggests that, at least for the four tRNA species investigated, reliable charging levels can be obtained from RNA extracted by the hot phenol method.

10.1128/mbio.02805-22.2 FIG S2 Northern blots showing tRNA charging levels after extraction of RNA with hot or cold phenol. Download FIG S2, PDF file, 0.8 MB. Copyright © 2023 Prossliner et al. 2023 Prossliner et al. https://creativecommons.org/licenses/by/4.0/ This content is distributed under the terms of the Creative Commons Attribution 4.0 International license.

### RNA modifications in the target region of northern blot probes can lead to misrepresented tRNA levels.

**FIG 2:**
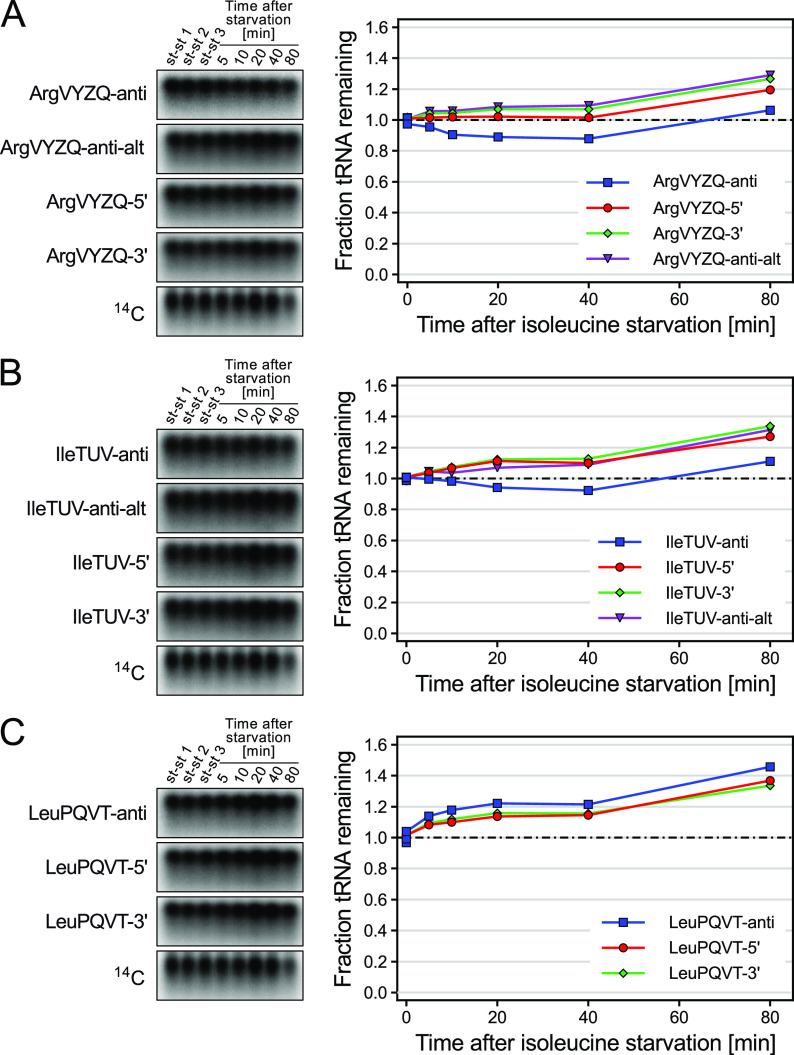
tRNA quantification can be influenced by the target region of northern blot probes. Northern blot analysis of tRNA^argVYZQ^ (A), tRNA^ileTUV^ (B), and tRNA^leuPQVT^ (C) levels in [^14^C]-labeled RNA samples obtained by hot phenol extraction after isoleucine starvation as described in Fig. 1. tRNAs were detected using oligonucleotide probes targeting different regions of each tRNA: anticodon, (tRNA-anti); 5′ terminus (tRNA-5′); 3′ terminus (tRNA-3′); anticodon, alternative (tRNA-anti-alt). Shown are the sections of northern blots corresponding to the target tRNA and the [^14^C]-signal of the tRNA fraction. The change in tRNA levels relative to the average of three steady-state (st-st 1 to 3) samples is plotted in the panels on the right. For normalization, the [^14^C]-signal of the entire tRNA fraction, which was confirmed to be stable in Fig. 1, was used. See Fig. S2 for the modification patterns of the selected tRNAs and the targeting regions of probes.

Although the tRNA fraction as a whole appeared to be stable when using hot phenol extraction (Fig. 1G), we initially observed a decrease of tRNA^argVYZQ^ and tRNA^ileTUV^ upon starvation when measuring a number of individual tRNAs by northern blotting (Fig. 2A and B, blue lines). To validate this observation, we repeated the quantification using alternative probes complementary to the 5′- and 3′ ends of the target tRNAs (see Text S1 and Fig. S3 for sequences and target regions, respectively). Strikingly, for both tRNAs the 5′ and 3′ probes yielded almost identical levels that were different from the probe complementary to the anticodon region of the tRNA (Fig. 2A and B, red and green lines). Importantly, this was not the case for tRNA^leuPQVT^, for which all probes yielded comparable results (Fig. 2C). The anticodon region of tRNAs is highly variable and therefore often used as the target region for oligonucleotide probes. However, tRNAs are also heavily modified in the anticodon stem-loop and its adjacent regions (reviewed in reference 17). Upon closer inspection, we noted that tRNA^argVYZQ^ and tRNA^ileTUV^ contain a large 3-(3-amino-3-carboxypropyl)uridine (acp3U) modification in the complementary region of the anticodon targeting probes (Fig. S3A and B) that is not present in tRNA^leuPQVT^ and the other tested tRNAs (Fig. S3C). We hypothesize that tRNA modifications on tRNA^argVYZQ^ and tRNA^ileTUV^ affected the hybridization temperature of the oligonucleotide probes used for detection of tRNA, a property that has previously been exploited for the detection of RNA modifications (18). Indeed, when the target region of the anticodon probe was shifted away from acp3U (Fig. S3A and B), apparent tRNA levels were comparable to those measured with the 5′ and 3′ probes (Fig. 2A and B, purple lines). This analysis uncovers another pitfall in the quantification of tRNAs that may lead to underestimation of tRNA levels in conditions in which *de novo* tRNA synthesis is halted and the pool of hypomodified tRNAs is depleted as the existing tRNAs are matured, namely, the risk that probes may hybridize with uneven efficiencies to hypomodified and mature tRNAs. We remark that the highly modified nature of tRNA is known to pose a significant obstacle for reverse-transcription-based quantification approaches (i.e., reverse transcription-quantitative PCR [RT-qPCR] and transcriptome sequencing [RNA-seq]) due to pausing or stopping of reverse transcriptase at modified nucleotides (19–21). In principle, relative tRNA levels could be estimated by methods that rely on reverse transcriptase, but only if all samples contain the same ratio of hypomodified to fully modified tRNAs. In our experience, the number of reads mapping to tRNA genes from deep RNA sequencing of samples harvested just 5 and 10 min after amino acid starvation was dramatically reduced relative to samples from the same culture immediately prior to starvation (12, 22). We presume the dramatic decline in tRNA reads after starvation is due to rapid depletion of the pool of *de novo* synthesized, hypomodified tRNA, when rRNA and tRNA transcription is strongly reduced.

10.1128/mbio.02805-22.3 FIG S3 RNA modification pattern of tRNAs that may influence probe binding. Download FIG S3, PDF file, 0.5 MB. Copyright © 2023 Prossliner et al. 2023 Prossliner et al. https://creativecommons.org/licenses/by/4.0/ This content is distributed under the terms of the Creative Commons Attribution 4.0 International license.

10.1128/mbio.02805-22.10 TEXT S1 Supplemental methods and references. Download Text S1, PDF file, 0.6 MB. Copyright © 2023 Prossliner et al. 2023 Prossliner et al. https://creativecommons.org/licenses/by/4.0/ This content is distributed under the terms of the Creative Commons Attribution 4.0 International license.

### tRNAs levels remain stable during short-term amino acid starvation.

**FIG 3:**
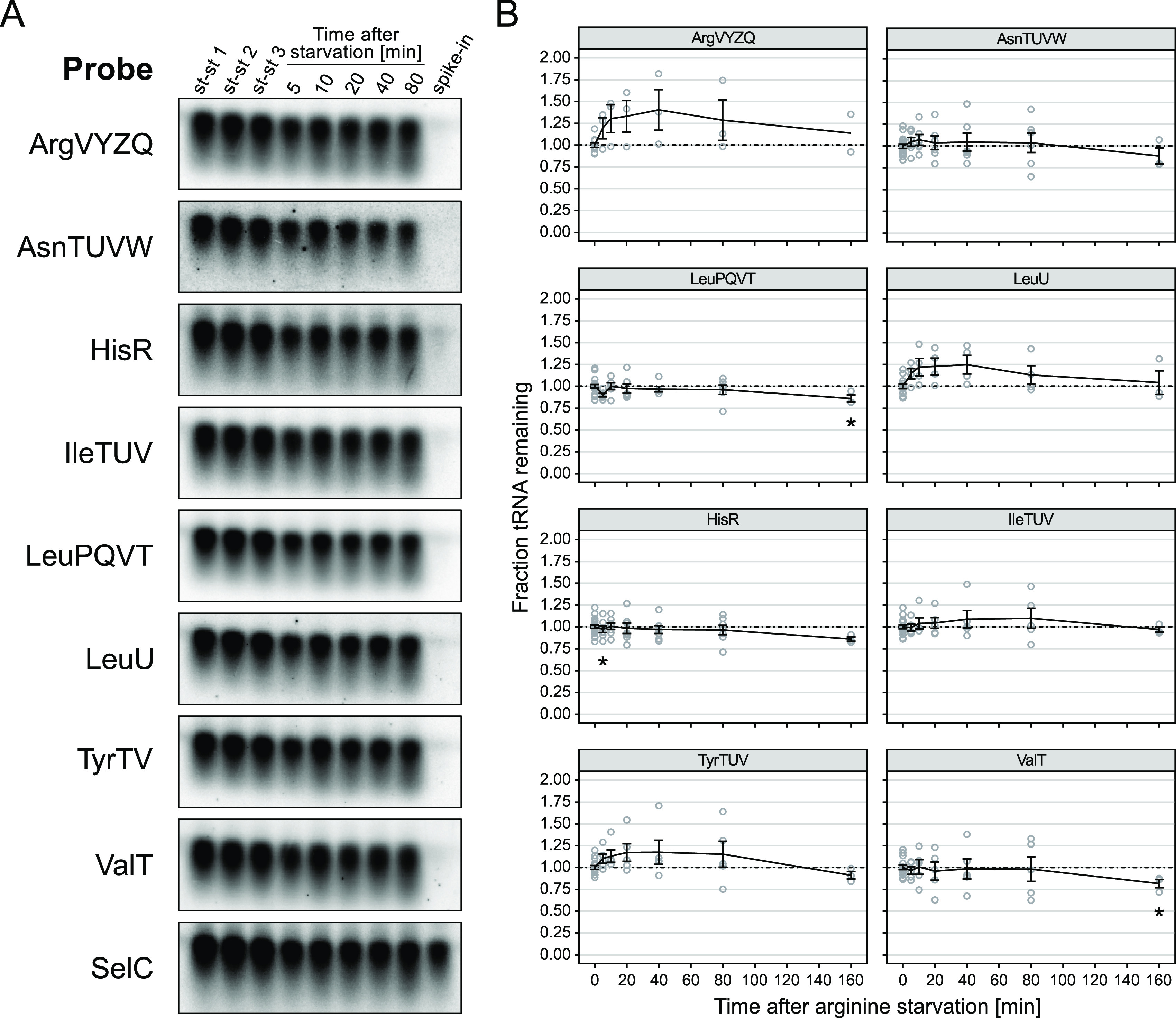
tRNAs are stable during short-term arginine starvation. (A) Northern blot analysis of selected tRNAs before and during arginine starvation. Culture samples of the arginine-auxotroph strain MAS1190 were taken during balanced growth (st-st 1 to 3) and during arginine starvation, which was induced by filtration and resuspension in medium lacking arginine. After addition of a small volume of spike-in cells expressing tRNA^selC^, total RNA was extracted by hot phenol extraction and separated by agarose gel electrophoresis. In addition to the test samples, a sample of RNA extracted from spike-in cells was included (spike-in) to estimate the amount of the target tRNA originating from endogenous expression in the spike-in culture. The RNA was transferred to a membrane by capillary transfer, and target tRNAs were detected by hybridization with specific [^32^P]-labeled DNA oligonucleotide probes. Shown are tRNA sections of an exemplary northern blot probed for a selection of tRNAs. (B) Quantification of tRNA levels after arginine starvation. tRNAs detected by northern blotting as exemplified in panel A were quantified by densitometry, and the fraction of tRNA remaining after starvation was calculated relative to three steady-state samples and normalized by the tRNA^selC^ signal originating from the spike-in. Lines represent the mean of at least three biological replicates (except for tRNA^argVYZQ^ at *t* = 160 min, where *n* = 2). Error bars indicate the standard error of the mean (SEM). Individual data points of replicates are shown as circles. *, *P* < 0.05 as determined by a two-tailed Student’s *t* test assuming unequal variances. The dash-dotted line indicates the steady-state tRNA level. See Fig. S4A for quantification of additional tRNAs.

Based on our evaluation of extraction methods, we proceeded to systematically investigate tRNA levels after amino acid starvation using hot phenol as our routine RNA extraction method. We applied a previously described method of data normalization, in which a small, fixed amount of a whole-cell spike-in culture, expressing large amounts of the rare tRNA^selC^, is added to each sample prior to RNA extraction. Levels of the specific tRNAs detected on the northern blot are then quantified relative to the levels of tRNA^selC^ detected in the same lane of the blot to normalize for sample-to-sample variations in RNA extraction efficiency and gel loading (3, 22). For the purpose of comparing RNA levels in cells harvested under different growth conditions, the whole-cell spike-in method of normalization is preferable to methods that rely on normalization to an endogenous reference RNA or to total RNA in the samples, because it does not rely on any assumptions regarding the RNA content of starved versus growing cells. Having overcome pitfalls in tRNA quantification related to the RNA extraction protocol, the normalization method and the probe design, we quantified a selection of tRNAs during arginine starvation, using an arginine auxotroph E. coli strain. As shown in Fig. 3 and Fig. S4A, the levels of most tested tRNAs either remained constant or increased slightly during the starvation period. The only observed significant exceptions were a transient drop of tRNA^hisR^ by 10% at 5 min and a 14% and 18% decrease of tRNA^leuPQVT^ and tRNA^valT^, respectively, after 160 min of starvation. However, as discussed below, this trend did not continue when starvation times were increased. Thus, there is no net loss of tRNAs under amino acid starvation. The observation appeared to be independent of the amino acid that was starved for, as leucine starvation showed comparable results (Fig. S4B). Upon amino acid starvation, tRNA and rRNA transcription is inhibited by the alarmone (p)ppGpp synthesized by the stringent factor RelA (23). To confirm that we would detect the expected changes in tRNA levels using the improved approach, we determined tRNA levels in a *relA*^–^ mutant (SAA21) after arginine starvation (Fig. S5). In this mutant, the levels of all nine tested tRNA species continued to increase in abundance after the onset of starvation in the *relA*^–^ mutant, with the increase ranging from approximately 1.5-fold (tRNA^argVYZQ^, tRNA^leuU^) to approximately 2.4-fold (tRNA^valT^) after 160 min of starvation. Consistent with this, treatment of cells with chloramphenicol, a ribosome-targeting antibiotic that blocks translation and is known to stimulate rRNA and tRNA synthesis by suppression of (p)ppGpp synthesis (24–26), resulted in a similar increase of tRNA levels (Fig. S6A).

10.1128/mbio.02805-22.4 FIG S4 tRNAs are stable during short-term arginine and leucine starvation. Download FIG S4, PDF file, 0.5 MB. Copyright © 2023 Prossliner et al. 2023 Prossliner et al. https://creativecommons.org/licenses/by/4.0/ This content is distributed under the terms of the Creative Commons Attribution 4.0 International license.

10.1128/mbio.02805-22.5 FIG S5 tRNA levels increase after arginine starvation in a *relA^–^* strain. Download FIG S5, PDF file, 0.7 MB. Copyright © 2023 Prossliner et al. 2023 Prossliner et al. https://creativecommons.org/licenses/by/4.0/ This content is distributed under the terms of the Creative Commons Attribution 4.0 International license.

10.1128/mbio.02805-22.6 FIG S6 tRNAs increase or are stable during chloramphenicol-induced repression of the stringent response, phosphate starvation, and rifampicin treatment. Download FIG S6, PDF file, 0.8 MB. Copyright © 2023 Prossliner et al. 2023 Prossliner et al. https://creativecommons.org/licenses/by/4.0/ This content is distributed under the terms of the Creative Commons Attribution 4.0 International license.

### tRNA levels are stable during short-term phosphate starvation.

**FIG 4:**
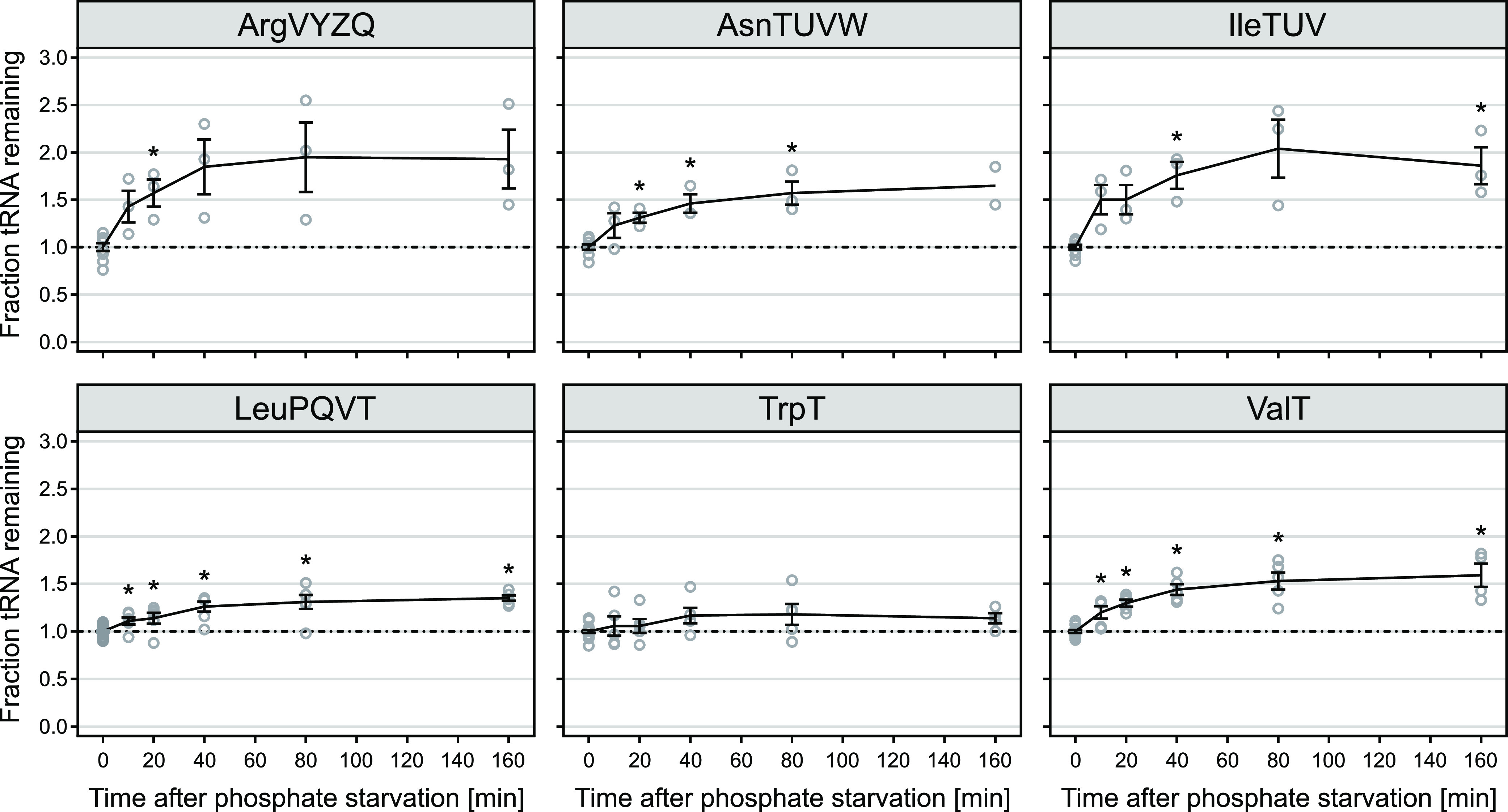
tRNAs are not degraded during short-term phosphate starvation. Levels of selected tRNAs in MAS1081 after phosphate starvation were determined by northern blotting analysis as described in Fig. 3. Lines represent the mean of at least three biological replicates (except for tRNA^asnTUVW^ at *t* = 160 min, where *n* = 2). Error bars indicate the SEM. Individual data points of replicates are shown as circles. *, *P* value < 0.05 as determined by a two-tailed Student’s *t* test assuming unequal variances. The dash-dotted line indicates the steady-state level. See Fig. S6B for measurements of an additional tRNA (tRNA^tyrTV^).

Having confirmed that tRNA levels are not reduced during amino acid starvation, we sought to test tRNA stability under other conditions that limit protein synthesis. The half-lives of ribosomal RNAs 16S and 23S are reduced upon starvation for a variety of nutrients, including isoleucine, glucose, phosphate, and nitrogen (2, 3, 6, 7, 27, 28). Since tRNAs and rRNAs are coregulated at the level of synthesis, we reasoned that their degradation might also occur under the same conditions. We have shown previously that in short-term starvation conditions, phosphate deprivation had a strong effect on rRNA integrity (3). Therefore, we measured tRNA levels during early phosphate starvation. As shown in Fig. 4 and Fig. S6B, we did not detect any loss of tRNA during phosphate starvation. In fact, the levels of several tRNAs increased in the first 40 to 80 min of starvation before reaching a plateau at up to 1.9-fold of the steady-state level (tRNA^ileTUV^).

### tRNAs are stable during rifampicin treatment.

**FIG 5:**
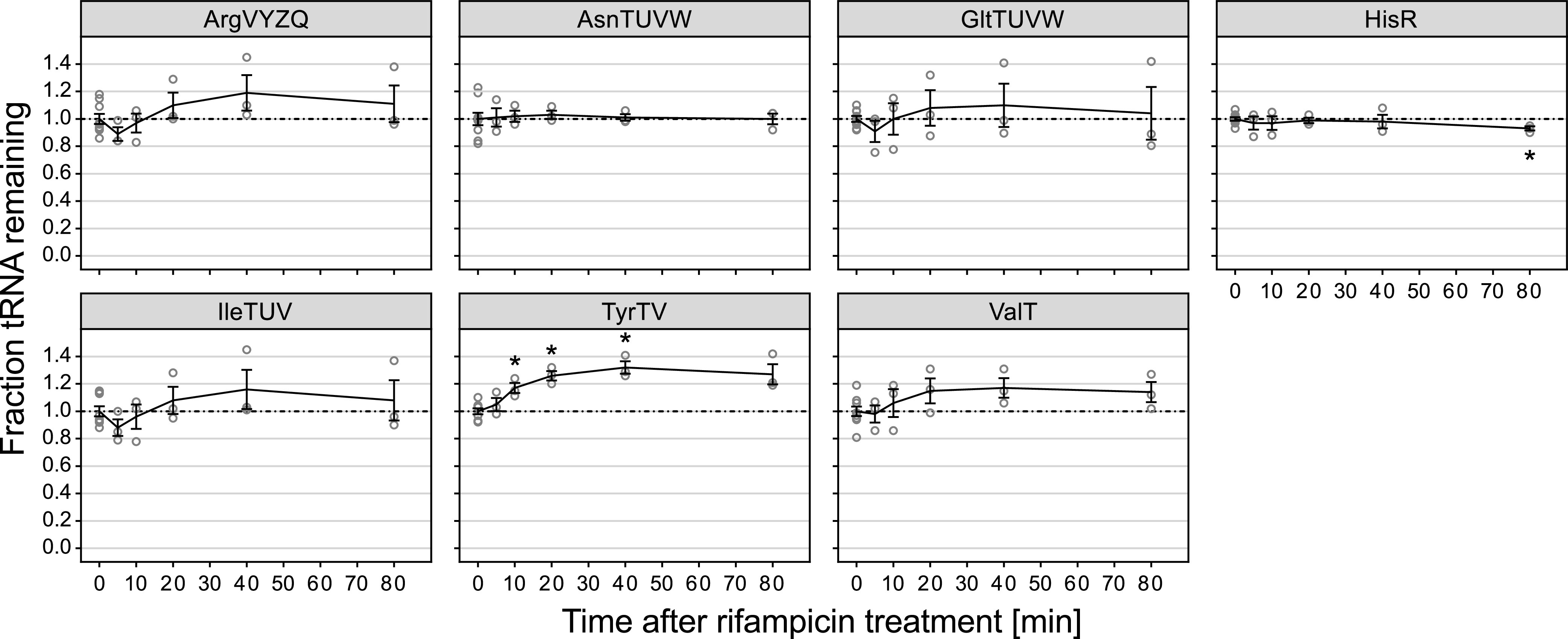
tRNAs are stable during rifampicin-induced transcription arrest. Levels of selected tRNAs after rifampicin treatment were determined by northern blotting analysis as described in Fig. 3. Rifampicin was added to steady-state cultures at time zero, followed by incubation for 80 min. Lines represent the mean of three or more biological replicates. Error bars indicate the SEM. Individual data points of replicates are shown as circles. *, *P* < 0.05 as determined by a two-tailed Student’s *t* test assuming unequal variances. The dash-dotted line indicates the steady-state level. See Fig. S6C for quantification of additional tRNAs.

Both amino acid (Fig. 3; Fig. S4) and phosphate starvation (Fig. 4; Fig. S6B) not only showed that tRNA levels remained stable but, in fact, resulted in a slight increase of tRNA levels after starvation compared to their level immediately prior to the onset of starvation. Our measurements of net tRNA levels do not reveal whether tRNAs are, in fact, stable, or if continued tRNA synthesis after starvation might compensate and mask cooccurring tRNA degradation. Therefore, we expanded our analysis to treatment with the transcription inhibitor rifampicin. We reasoned that if a tRNA-degradation mechanism was in place under conditions that decrease the rate of protein synthesis, abolishing *de novo* RNA synthesis by transcription inhibition would result in a decrease of the existing pool of tRNAs. However, as shown in Fig. 5 and Fig. S6C, tRNA levels remained largely constant after rifampicin treatment, showing that at least under this condition, there was no degradation of existing tRNAs. Surprisingly, tRNA^tyrTUV^ levels appeared to increase to 1.3-fold relative to the level prior to addition of rifampicin (Fig. 5). Additional experiments are needed to clarify whether tyrosyl-tRNA is in fact transcribed by the rifampicin-inhibited RNA polymerase or whether the apparent increase can be explained by another change in this particular tRNA, such as an altered modification pattern.

### tRNA levels are stably maintained during long-term nutrient starvation.

**FIG 6:**
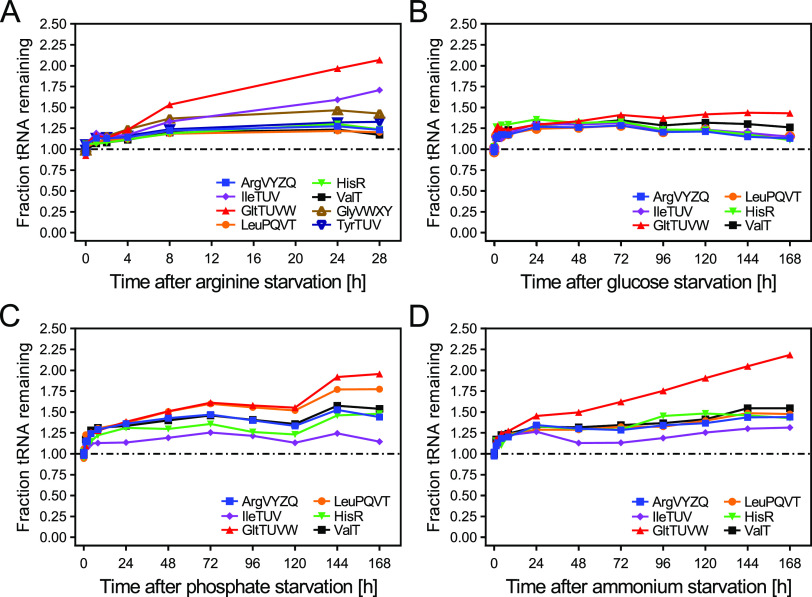
tRNAs are stable during long-term starvation for a variety of nutrients. (A) Levels of selected tRNAs during long-term arginine starvation of MAS1190. Starvation was induced by filtration and resuspension in the same medium lacking arginine. The mean of two independent replicates is shown. Error bars have been omitted for the sake of clarity. Data from individual replicates are shown in Fig. S7A. The dash-dotted line indicates the steady-state level in this and all following panels. (B to D) Levels of selected tRNAs in MAS1081 during long-term starvation for the macronutrients glucose (B), phosphate (C), and ammonium (D). Starvation was induced by filtration and resuspension in medium lacking the respective nutrient. The mean of three independent biological replicates is shown. Error bars have been omitted for the sake of clarity. Data from three independent biological replicates, including statistical analysis, are shown in Fig. S7B to D.

Our inability to detect tRNA degradation during short-term starvation prompted us to investigate the extent of tRNA stability in long-term-starved cultures. We investigated tRNA levels for up to 28 h of arginine starvation and up to 168 h of starvation for glucose, phosphate, or ammonium. As shown in Fig. 6A and Fig. S7A, tRNA levels consistently increased during long-term arginine starvation, albeit to different extents depending on the tested tRNA. A similar trend was observed during starvation for the major macronutrients glucose, phosphate, and ammonium (Fig. 6B to D; Fig. S7B to D): all tested tRNAs showed an initial increase in the first 8 h of starvation and remained stable or gradually increased for the remainder of the experiment. Of the tRNAs that continued to accumulate throughout the experiment, tRNA^gltTUVW^ showed the most pronounced increase, in particular, during arginine and nitrogen starvation (Fig. 6A and D; Fig. S7A and D). When starvation was induced by depletion of nutrients rather than filtration and resuspension in starvation medium, tRNAs were similarly stable (Fig. S8A to C). Reports have shown that tRNA fragments accumulate during the stationary phase in E. coli and Salmonella enterica (5, 29). To ensure that the relatively low resolution of agarose gels used for northern blotting did not lead us to include tRNA fragments in the band quantified for full-length tRNAs, we analyzed a set of long-term samples by high-resolution denaturing PAGE and northern blotting. Although we could detect tRNA halves, the fraction was negligible for quantification of intact tRNAs (Fig. S9A and B). In addition, when we labeled total cellular RNA using [^14^C]-uracil as described above (Fig. 1A) before long-term starvation for the macronutrients, the tRNA fraction on the resulting northern blots appeared stable in all three conditions for up to 7 days of starvation (Fig. S9C to E). This confirms that there is no net loss of tRNAs during long-term starvation. In summary, these results show that tRNA levels are stably maintained during prolonged nutrient starvation and suggest that E. coli may even commit resources to continue a low level of tRNA transcription for several days of starvation.

10.1128/mbio.02805-22.7 FIG S7 Biological replicates of tRNA quantification after starvation for arginine, glucose, phosphate, or ammonium. Download FIG S7, PDF file, 0.6 MB. Copyright © 2023 Prossliner et al. 2023 Prossliner et al. https://creativecommons.org/licenses/by/4.0/ This content is distributed under the terms of the Creative Commons Attribution 4.0 International license.

10.1128/mbio.02805-22.8 FIG S8 tRNA and rRNA levels after starvation induced by depletion of glucose, phosphate, or ammonium. Download FIG S8, PDF file, 0.6 MB. Copyright © 2023 Prossliner et al. 2023 Prossliner et al. https://creativecommons.org/licenses/by/4.0/ This content is distributed under the terms of the Creative Commons Attribution 4.0 International license.

10.1128/mbio.02805-22.9 FIG S9 Verification of tRNA and rRNA quantification during long-term starvation for glucose, phosphate, or ammonium. Download FIG S9, PDF file, 0.6 MB. Copyright © 2023 Prossliner et al. 2023 Prossliner et al. https://creativecommons.org/licenses/by/4.0/ This content is distributed under the terms of the Creative Commons Attribution 4.0 International license.

**FIG 7:**
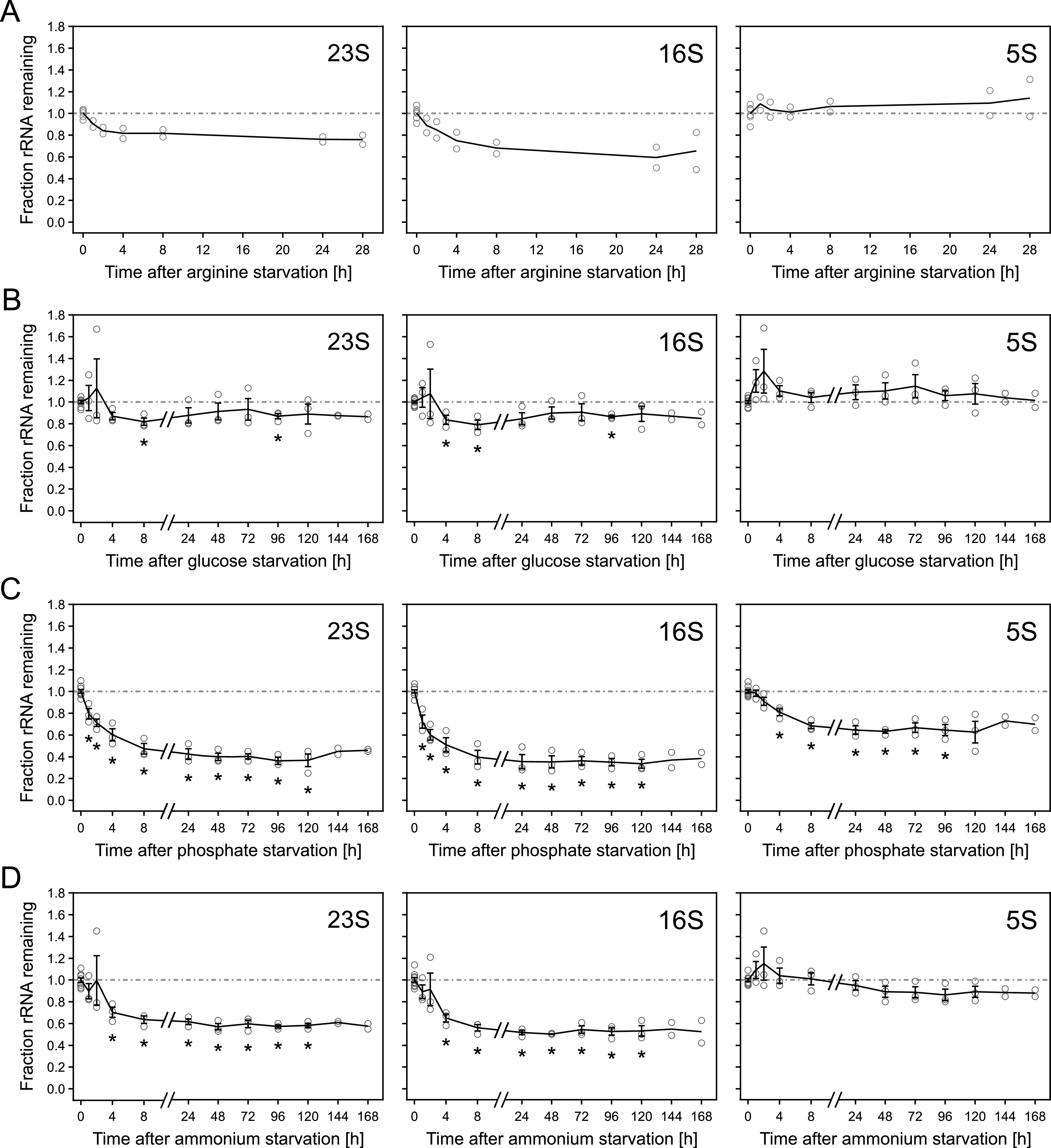
rRNA degradation varies between starvation conditions and species of rRNA. (A) Levels of rRNAs during long-term arginine starvation of MAS1190 carried out as described in Fig. 6A. Circles represent data points of two independent biological replicates; lines indicate the mean of the replicates. (B to D) Levels of rRNAs in MAS1081 during long-term starvation for glucose (B), phosphate (C), and ammonium (D). Lines represent the mean of three replicates except for *t* = 144 to 168 h, where *n* = 2. Error bars indicate the SEM. Individual data points of replicates are shown as circles. *, *P* < 0.05 as determined by a two-tailed Student’s *t* test assuming unequal variances. Note that the *x* axis is split into two segments to ensure sufficient distinction of the data points up to 8 h.

Intriguingly, while tRNA levels remained stable, we observed degradation of ribosomal RNAs. Both 23S and 16S rRNA levels decreased during all tested starvation conditions, while 5S rRNA was only affected by phosphate starvation. We noted that the rRNAs stabilized at different levels depending on the type of nutrient the cells were starved for, with the most extensive degradation occurring during phosphate starvation, followed by ammonium starvation, while we only observed minor adjustments to the rRNA pool in response to glucose or arginine starvation. These observations were independent of whether starvation was induced by filtration or depletion (Fig. 7A to D; Fig. S8D to F). The rRNA levels also decreased when a lysis step was added before RNA extraction from long-term-starved cells (Fig. S9F to H), corroborating that the drop in rRNA levels was not due to possible effects of long-term starvation on RNA extraction efficiency. In addition, we observed accumulation of various fragments derived from [^14^C]-uracil-labeled rRNA after prolonged starvation (Fig. S9C to E), consistent with previous observations that rRNA is increasingly fragmented and degraded during long-term starvation for different nutrients (4, 5, 30).

**FIG 8:**
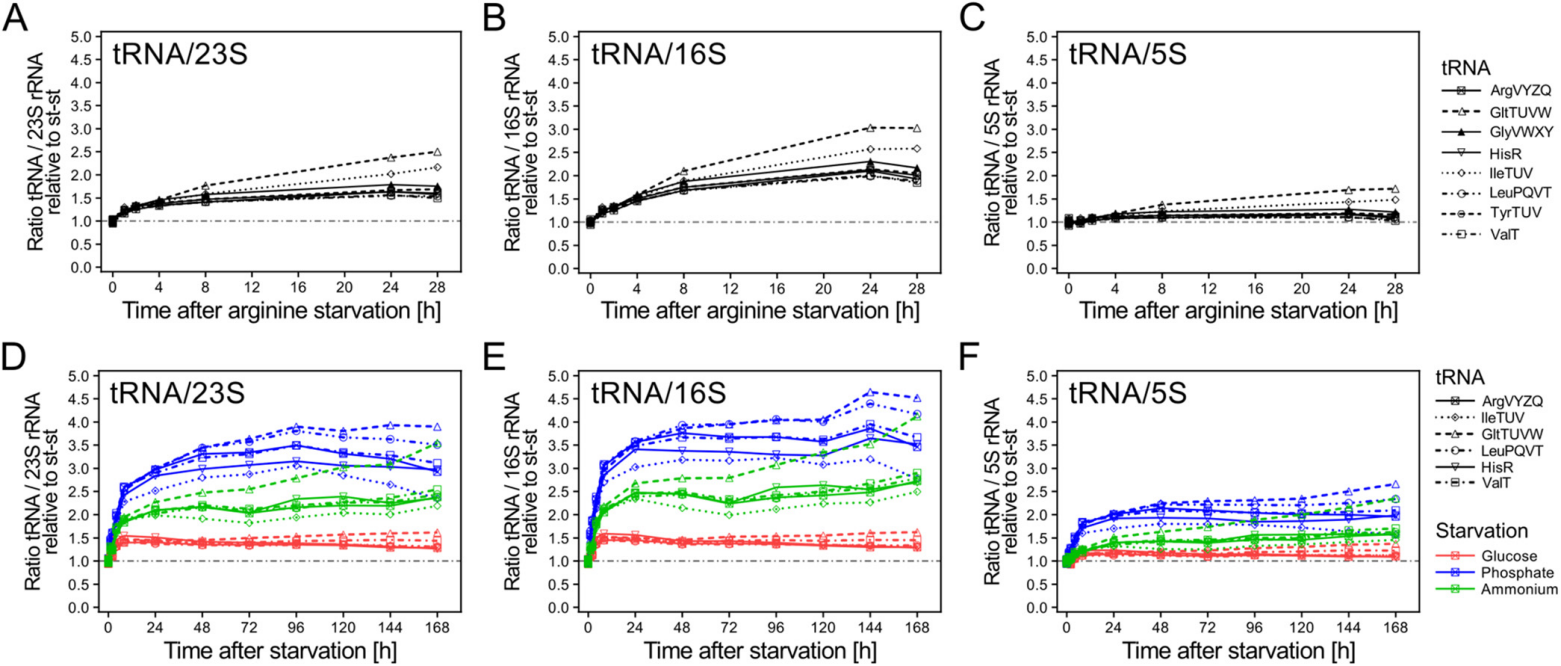
Differential stability of tRNAs and rRNAs during starvation leads to an excess of tRNAs. (A to C) Ratios of selected tRNAs to 23S (A), 16S (B), and 5S (C) rRNA during long-term arginine starvation. Ratios were calculated from band intensities of the respective tRNA/rRNA and normalized to the steady state ratio. The dash-dotted line indicates the steady-state level in these and the following panels. (D to F) Ratios of selected tRNAs to 23S (D), 16S (E), and 5S (F) rRNA during long-term glucose (red), phosphate (blue), and ammonium (green) starvation.

Taken together, our results illustrate that while tRNA and rRNA levels are believed to be largely coregulated at the level of their synthesis, their degradation kinetics appear uncoupled, resulting in highly increased tRNA/rRNA ratios in cultures starved for phosphate or nitrogen relative to cultures in balanced growth (Fig. 8).

## DISCUSSION

Here, we present an in-depth reassessment of E. coli tRNA levels under a variety of starvation conditions. This analysis corrects our previous model, in which we proposed that rapid tRNA degradation was triggered under conditions that reduce protein synthesis (12). The current study was motivated by our inability to identify factors involved in the proposed tRNA degradation pathway (S. Agrawal and T. Prossliner unpublished data; 10, 12). Using an improved and validated protocol for tRNA quantification, we report that tRNA levels in fact remained virtually unchanged upon amino acid starvation, regardless of whether cells were starved for isoleucine, arginine, or leucine (Fig. 2 and 3; Fig. S4). We extended our analysis to include short-term phosphate starvation and rifampicin-induced transcription arrest, as well as long-term amino acid, glucose, phosphate, and ammonium starvation. Remarkably, in all of the tested conditions tRNA levels remained constant or increased, corroborating that tRNAs truly are stable RNAs (Fig. 3 to 6). In contrast, rRNA levels decreased substantially during starvation (Fig. 7). This resulted in considerable starvation-induced changes in the ratio between tRNAs and rRNAs that were dependent on the individual tRNA and rRNA and the nutrient for which the cells were starved (Fig. 8).

The observation that there was no net loss of tRNA from cultures under the tested conditions does not exclude the possibility that some degree of tRNA degradation occurred but was outweighed by simultaneous tRNA synthesis. However, the results of our radiolabeling approach show that tRNAs synthesized during exponential growth (i.e., when the labeling took place) remained stable during early isoleucine starvation (Fig. 1F and G). In addition, transcription arrest by rifampicin treatment did not result in a decrease of existing tRNAs (Fig. 5), demonstrating that continued tRNA transcription is not required to maintain the nearly unchanged tRNA levels under this condition. This is in agreement with early studies on the stability of tRNAs during isoleucine (31), phosphorus, carbon, and nitrogen starvation (27). More recent work on the E. coli response to oxidative stress came to a similar conclusion and, instead, proposed that a specific tRNA (tRNA^gly^) is inactivated rather than degraded (32), although other studies have reported extensive degradation of tRNAs during oxidative stress (33, 34). These opposing reports and our own results underscore that the seemingly trivial task of quantifying an abundant RNA species such as tRNA is not straightforward for cells grown outside the steady-state growth regime and can contain hidden pitfalls at every step of the analysis, from RNA extraction to target detection and normalization.

As demonstrated by our RNA labeling approach, cold phenol extraction yields mostly tRNA (Fig. 1F), which for some applications such as measurements of tRNA charging levels, and in principle also for tRNA quantification, can be desirable. However, our analysis showed that extraction is strongly influenced by the physiological state of the cell, and both overall extraction efficiency and recovery of individual RNA species dropped substantially after starvation (Fig. 1D and 1H, respectively). Similarly, the widely used TRI Reagent protocol exhibits severe extraction biases. The method appears to preferentially extract short RNAs even from E. coli cultures in balanced growth (Fig. 1F). When RNA from starved cells was extracted by TRI Reagent, the [^14^C/^3^H] ratio was indistinguishable from that of the samples harvested from balanced cultures (Fig. 1E). However, analysis of individual RNA species showed a clear growth-state bias for the 23S, 16S, and 5S rRNA species, which were extracted with reduced efficiency from the starved cells (Fig. 1I). We did not detect a growth-state effect on the extraction of the tRNA pool with TRI Reagent, which explains the growth-phase-insensitive [^14^C/^3^H] ratio since tRNAs dominated the total RNA that could be recovered with TRI Reagent. We note that since extraction of 5S rRNA, which is 120 nucleotides (nt) in length and thus only slightly larger than some tRNAs (e.g., tRNA^serV^, 93 nt) was strongly reduced after starvation, it cannot be excluded that TRI Reagent extraction of individual, larger tRNAs would be sensitive to the growth state of the cultures. Based on these results, we concluded that the methods used in our previous work did not reliably reflect the actual distribution of RNAs in the cell, and importantly, were strongly affected by starvation. Hot phenol extraction, on the other hand, resulted in an increased overall yield (Fig. 1B) that only decreased to the extent expected from starvation-induced degradation of rRNA (3, 35). We therefore opted for hot phenol extraction as our routine method for the reassessment of tRNA stability presented in the second part of this work.

We can only speculate about the reasons behind the severely decreased RNA extraction efficiency of certain extraction methods after amino acid deprivation. Bacterial cells undergo extensive morphological changes upon transition to growth arrest, including rearrangements in the composition of cell membranes and cell wall that are thought to contribute to increased resistance to a variety of stresses (reviewed in reference 1). These morphological changes may render the cells refractory to both mechanical stress (i.e., vortexing) and the action of chaotropic components of lysis solutions (e.g., phenol, guanidine thiocyanate) which are the basis of common RNA extraction protocols. Early microscopy studies have documented a rapid increase of cell wall thickness of Enterococcus faecalis cells after amino acid deprivation (36). It has also been shown that amino acid starvation leads to rapid changes of the cell wall composition of E. coli that almost entirely abolish penicillin-induced autolysis within 15 min of starvation (37). Importantly, the same study showed that lysis efficiency by treatment with TCA or repeated freeze-thaw cycles gradually decreases after starvation, albeit at a slower rate than penicillin-induced lysis. In our earlier study we also observed reduced extraction of tRNA with cold phenol after rifampicin treatment of unstarved cells (12), while we show in this work that tRNA levels remain stable after rifampicin treatment (Fig. 5). Apart from a recent report showing a rifampicin-induced decrease in cell size (38) little is known about morphological changes occurring in E. coli after rifampicin exposure. However, treatment of Staphylococcus aureus with supra-MICs results in extensive thickening of the cell wall (39), and a similar effect has been observed when treating S. aureus with tetracycline (40). If such changes are induced in E. coli, this could explain the reduced extraction efficiency of RNA from rifampicin-treated cells. Interestingly, treatment of E. coli with chloramphenicol did not alter the thickness of the cell wall (41), which is consistent with our observation that tRNA levels increased after chloramphenicol treatment regardless of which extraction method was used (Fig. S6A; reference 12). Our data also suggest that the effect of rifampicin on the extractability of RNA by cold phenol is independent of *de novo* protein synthesis. We saw a drastic loss of tRNA recovered by the cold phenol method even when rifampicin was added after 80 min of chloramphenicol treatment (12), while we could not detect any loss using hot phenol extraction (Fig. S6A).

The present study focused solely on the quantification of tRNAs in the Gram-negative bacterium E. coli. However, the finding that the very first step of the analysis, RNA extraction, can suffer from such severe bias in general and size-specific efficiency when growth conditions are changed is rather alarming. The growing interest in the bacterial stress responses and the physiology of growth arrest has brought about a plethora of studies analyzing whole transcriptomes and stable RNAs and other noncoding RNAs under such conditions, all depending on reliable extraction of RNA from a variety of sometimes notoriously resilient microorganisms. It can therefore not be emphasized enough that the feasibility of a chosen extraction method must be assessed before embarking on potentially costly and time-consuming downstream analyses. If, as in this case, the overall efficiency of the chosen extraction method is influenced by the experimental conditions, any quantification relative to the original condition will inevitably be skewed. Perhaps less obvious, but equally problematic, is a possible size bias during extraction.

Using a validated approach, we demonstrated remarkable stability of tRNAs under conditions that induce the stringent response, including prolonged starvation conditions (Fig. 6). This finding underscores that tRNA charging level rather than tRNA concentration is the most relevant parameter to measure in studies of the E. coli stringent response (42).

Prolonged nutrient starvation has repeatedly been shown to lead to extensive degradation of ribosomes and rRNA (4, 27, 30) through dedicated degradation pathways (2, 6, 7). Our findings therefore imply that degradation of the RNA components of the translation machinery is not an orchestrated mechanism that leads to a synchronized reduction of all components. Instead, the ratio between intact rRNAs and tRNAs is reduced when cells encounter nutrient starvation, to different extents depending on the type of nutrient starved for and even dependent on the particular rRNA and tRNA in question (Fig. 8). Considering that a large fraction of ribosomes are in an inactive, hibernating state during starvation (43), the balance between active ribosomes and available tRNAs would be shifted even further toward an excess of tRNAs upon starvation than what is revealed by RNA quantification. It remains to be determined whether this altered ratio constitutes a physiological advantage for the cell. Certainly, since translation is believed to operate at a rate close to the diffusion limit for the ternary complexes in nonstarved cells (44), maintenance of the concentration of tRNAs during starvation would ensure that at least the tRNA component of the ternary complexes remains at a sufficiently high concentration to support the remaining active ribosomes in synthesizing proteins at a reasonable elongation rate. Maintaining tRNAs may also ensure a rapid return to growth when nutrients are replenished. Although tRNAs are short RNA molecules in comparison to 23S and 16S rRNA, *de novo* synthesis, processing, and modification of the entire set of tRNAs needed for an efficient restart of translation could represent a bottleneck slowing down reentry into exponential growth and therefore lead to decreased fitness. This would implicate a delicate trade-off between degradation and maintenance of the individual RNA components of the translation machinery in which longer RNAs (i.e., 23S and 16S) are degraded, while short RNAs (5S and tRNAs) are not.

Notably, tRNA^gltTUVW^ consistently reached the highest level after prolonged starvation for various nutrients. While it is generally assumed that tRNA and rRNA synthesis is coregulated, tRNA^gltTUVW^ has been shown to be transcribed during the stationary phase from an internal promoter of the *rrn* operons containing *gltTUVW* (45). This could explain the continuous accumulation of tRNA^gltTUVW^ we observed, in particular, during arginine and nitrogen starvation (Fig. 6A to D). Whether this effect is of physiological importance remains to be determined.

In conclusion, our comprehensive analysis of tRNA stability in a variety of stress conditions, including nutrient starvation and antibiotic treatment, shows that these small RNAs are perhaps the most stable among the so-called stable RNAs. The preceding reevaluation of our prior approach led to the uncovering of severe biases in common RNA extraction and quantification methods with implications that reach far beyond the quantification of tRNAs.

## MATERIALS AND METHODS

### Bacterial strains and plasmids.

The bacterial strains used in this study are listed in Text S1. MAS1081 (E. coli K-12 MG1655 *rph*^+^ *gatC*^+^ *glpR*^+^) (3) served as the wild-type strain. For amino acid starvation experiments, arginine-leucine auxotroph MAS1190 (MAS1081 *ΔargG ΔleuA ΔpyrE*::*tetR*) was used. SAA21 (MAS1190 Δ*relA*::*kanR*) was generated by P1 transduction using CF1651 (*ΔrelA251*::*kanR*) (46) as the donor strain. MAS1074, a BL21(DE3) derivative carrying pET11a(*selc*) that produces high amounts of the rare tRNA^selC^ upon IPTG (isopropyl-β-d-thiogalactopyranoside) induction was used as the spike-in strain for normalization of tRNA quantification.

### Media and growth condition.

For cloning and recombination, cells were grown in yeast extract-tryptone (YT) medium at 37°C with shaking. For all physiological experiments, cells were cultured in MOPS (3-morpholinopropane-1-sulfonic acid) minimal medium (47) supplemented with 0.2% glucose as the carbon source at 37°C with shaking in a water bath. To ensure balanced growth, cultures were kept in the exponential phase for at least 10 generations before the start of the experiment. Bacterial growth was estimated by measuring the optical density at 436 nm (OD_436_). Starvation and antibiotic treatment were routinely performed when cultures reached an OD_436_ of 0.5 to 0.85.

### Determination of RNA extraction efficiency by ^14^C-labeling.

To determine the efficiency of common RNA extraction methods, total cellular RNA was radioactively labeled, and recovery of radioactively labeled RNA after purification was measured. An exponentially growing culture of MAS1081 of an OD_436_ of approximately 0.1 was supplemented with [2-^14^C]-uracil (0.1 μCi/μL; 53 mCi/mmol; Biotrend) to a final concentration of 1 μCi/mL (18.9 μM), and incubation continued until an OD_436_ of approximately 0.4 was reached, at which point unlabeled uracil (1 mg/mL stock solution) was added to a final concentration of 20 μg/mL (178 μM). At an OD_436_ of approximately 0.8 three steady-state samples (st-st 1 to 3) were taken. Immediately thereafter, isoleucine starvation was induced by addition of l-valine (50 mg/mL) to a final concentration of 400 μg/mL, and samples were taken at 5, 10, 20, 40, and 80 min after starvation. All samples were taken by transferring 4 to 6 mL of culture into a 1/5 volume of cold stop solution (5% phenol in ethanol) (3, 48) on ice. To serve as a spike-in for normalization, a culture of MAS1081 was grown in MOPS minimal medium supplemented with 20 μCi/mL (0.98 μM) [5-^3^H]-uridine (20.4 Ci/mmol; Perkin Elmer) and harvested by transfer to a 1/5 culture volume stop solution. To each sample, 0.625 mL of the stopped [^3^H]-labeled spike-in culture was added and mixed by inverting, and the stopped sample/spike-in mixture was divided for RNA purification using the methods described below. To determine the total incorporated radioactivity in the samples, 50 μL of the cell suspension or 5 μL of extracted RNA was precipitated with 0.5 mL of TCA (5%) for approximately 30 min at 0°C. In addition, to obtain an estimate for incorporation of [^14^C]/[^3^H] into DNA, identical samples were treated with 0.5 mL of NaOH (0.5 M) and incubated for 2 h at 37°C in a water bath before TCA precipitation. TCA precipitates were collected on glass fiber filters (Advantec) by vacuum filtration, washed three times with 5 mL ice-cold 5% TCA, and dried overnight at room temperature (RT) before the addition of 5 mL Ultima Gold scintillation cocktail (Perkin Elmer) and measurement of disintegrations per minute (dpm) by liquid scintillation counting (LSC).

Before calculating the efficiency of RNA extraction, dpm originating from radiolabeled DNA (NaOH-treated samples) were subtracted from all sample counts to obtain [^14^C] and [^3^H] counts solely originating from labeled RNA. The overall efficiency of RNA extractions was calculated as the fraction of RNA recovered by the tested extraction method relative to the total, TCA-precipitable RNA. Recovery of total RNA after isoleucine starvation relative to the steady state was calculated by normalizing all samples to the [^3^H] counts originating from spike-in cells and calculating the fraction of extracted RNA relative to the average RNA recovery of three steady-state samples (st-st 1 to 3).

### PAGE analysis of radiolabeled RNA.

Radiolabeled RNA obtained as described above was separated by denaturing polyacrylamide gel electrophoresis (PAGE) on 5% polyacrylamide gels buffered with 1× TBE (100 mM Tris Base, 100 mM boric acid, 2 mM EDTA) and supplemented with 8 M urea. Samples were diluted in loading buffer (95% formamide, 0.025% bromophenol blue, 0.025 xylene cyanol, 5 mM EDTA, 0.025% SDS) prior to loading. Electrophoresis was performed at 15 V/cm, and the resulting gels were fixed by soaking in 50% ethanol and 10% acetic acid, dried, and imaged by phosphor imaging. To determine relative levels of individual RNA species, corresponding bands were cut out, treated with RNase A in TE buffer, and subjected to scintillation counting. Recovery of individual RNA species after starvation was calculated by normalization to [^3^H] counts in the bands originating from spike-in cells as described for the calculation of total RNA extraction efficiency.

### Starvation and antibiotic treatment assays.

Starvation for arginine or leucine in auxotroph strains was induced by filtration of cell cultures at 37°C, followed by a wash with prewarmed MOPS minimal medium lacking the respective amino acid and resuspension in the initial volume of prewarmed MOPS minimal medium lacking the respective amino acid. Starvation for glucose, phosphate, or ammonium was induced by filtration of cell cultures at 37°C, followed by one wash with prewarmed MOPS starvation medium and resuspension in the initial volume of prewarmed MOPS starvation medium. Antibiotic treatment was performed by addition of rifampicin or chloramphenicol to a final concentration of 100 μg/mL.

### Cell harvest and addition of spike-in cells.

At the indicated time points cells were harvested by transferring 1.5 mL of culture to an Eppendorf tube containing 0.3 mL stop solution on ice. Stopped samples were kept on ice until all samples were harvested. In parallel, a spike-in culture was prepared by growing MAS1074 to an OD_436_ of approximately 0.1 and overexpressing tRNA^selC^ by induction with 1 mM IPTG for approximately 4 h before rapid cooling and storage of the culture on ice. A volume of MAS1074 spike-in culture corresponding to 2% of the OD_436_ units of the sample was added to each sample, and the cell mixture was harvested by centrifugation at 20,000 × *g* at 4°C for 2 min. The supernatant was removed, and total RNA was immediately extracted from cell pellets using hot phenol extraction as described below. For long-term experiments, the spike-in culture was rapidly cooled in an ice bath, thoroughly mixed with two volumes of RNA*later* (Invitrogen), and aliquoted before freezing in liquid nitrogen and storage at −80°C. Spike-in cells are stably kept in RNA*later* for at least 30 days (30). At each time point an aliquot was thawed on ice, mixed, and added to the cell samples taken on the same day before proceeding immediately to RNA extraction.

### Extraction of RNA.

**(i) Hot phenol (HP).** The following modified hot phenol extraction procedure was used for determination of RNA extraction efficiency as well as all tRNA quantification experiments presented here. In brief, cell pellets were resuspended in 200 μL ice-cold solution 1 (0.3 M sucrose, 0.01 M NaOAc, pH 4.5), vortexed, and then mixed with 200 μL solution 2 (2% SDS, 0.01 M NaOAc, pH 4.5; RT) by inverting. The tubes were placed at 65°C in a water bath for 1.5 min, and 400 μL preheated acidic phenol (pH 4.5) was added, followed by vortexing and incubation at 65°C for 3 min. Samples were rapidly frozen in liquid nitrogen for 15 s and immediately centrifuged at 20,000 × *g* at RT for 5 min. The upper phase was transferred to a fresh tube containing 200 μL preheated acidic phenol (pH 4.5), vortexed, and incubated at 65°C for 3 min. Samples were frozen in liquid nitrogen for 15 s and centrifuged at 20,000 × *g* at RT for 5 min. The upper phase was transferred to a new tube, and 50 μL NaOAc (3 M, pH 4.7) and 1 mL ethanol (96%) was added before incubation of at least 15 min at −20°C or overnight at −80°C. Precipitated RNA was collected by centrifugation at 20,000 × *g* at 4°C for 20 min, and the pellet was washed with 70% cold ethyl alcohol (EtOH) and subsequently dried at RT for 10 to 20 min. Finally, the RNA was dissolved in 50 μL RNA storage buffer (10 mM NaOAc, pH 4.7, 1 mM EDTA) and stored at −80°C.

**(ii) Hot phenol, modified (HP+).** The modified hot phenol extraction was performed as described above, with the exception of a lysis step at the beginning of the procedure, in which cell pellets were resuspended in 200 μL ice-cold solution 1 supplemented with 2% acidic phenol and lysed by sonication at 4°C using a Bioruptor Plus (Diagenode) equipped with a Bioruptor water cooler (Diagenode). Sonication was performed according to the manufacturer’s instructions at the high-power setting for 10 min in sonication cycles of 30 s on and 30 s off.

**(iii) Cold phenol (CP).** Cold phenol extraction was essentially performed as described in reference 13. Briefly, cell pellets were resuspended in 300 μL NaOAc (3 M, pH 4.7) and EDTA (10 mM); the mixture was transferred to a tube containing 300 μL phenol equilibrated with the same buffer and vortexed 10 times for 15 s with a cooling step on ice between each cycle. Samples were centrifuged at 20,000 × *g* at 4°C for 15 min, and the aqueous phase was transferred to a new tube containing 300 μL fresh phenol and vortexed 4 times for 15 s with a cooling step on ice between each cycle. Samples were centrifuged at 20,000 × *g* at 4°C for 10 min, and the aqueous phase was transferred to a new tube containing 750 μL ethanol (96%), mixed, and incubated overnight at −80°C. Precipitated RNA was collected by centrifugation at 20,000 × *g* at 4°C for 30 min, and the pellet was washed with 70% ice-cold ethanol and subsequently dried at RT for 10 to 20 min. Finally, the RNA was dissolved in 50 μL RNA storage buffer and stored at −80°C.

**(iv) TRI Reagent (TRI).** TRI Reagent extraction was performed according to the manufacturers protocol (Sigma-Aldrich, T9424). In brief, cell pellets were resuspended in 1.5 mL of TRI Reagent and incubated at RT for 5 min, followed by addition of 0.3 mL chloroform. The mixture was shaken vigorously for 15 s, incubated at RT for 15 min, and centrifuged at 12,000 × *g* at 4°C for 15 min. The aqueous phase was transferred to a new tube, and 0.75 mL of propan-2-ol was added and mixed in by repeated inversion of the tubes, followed by incubation at RT for 10 min. Samples were then centrifuged at 12,000 × *g* at 4°C for 10 min, the supernatant was removed, and the resulting RNA pellet washed by addition of 1.5 mL of 75% ethanol followed by centrifugation at 7,500 × *g* at 4°C for 5 min. The supernatant was removed, and the pellet was air-dried for 5 min and dissolved in 40 μL RNA storage buffer.

**(v) TRI Reagent, modified (TRI^+^).** The modified TRI Reagent extraction was performed as follows. Cell pellets were resuspended in 1 mL of TRI Reagent solution and incubated at RT for 5 min. Then, 200 μL chloroform was added, and the samples were vortexed for 15 s and incubated at RT for 5 min with occasional gentle mixing by inverting. Samples were centrifuged at 20,000 × *g* at 4°C for 15 min, and the aqueous, colorless phase was transferred to a fresh tube. Propan-2-ol (700 μL) was added, and the samples were incubated for 20 min at RT followed by centrifugation at 20,000 × *g* at 4°C for 20 min. The RNA pellet was washed in ethanol (70%), dried at RT for 10 to 20 min, dissolved in 40 μL RNA storage buffer, and stored at −80°C.

### Northern blot analysis of tRNA levels.

Denaturing formaldehyde agarose gel electrophoresis was performed as described in reference 3. In brief, 2% agarose gels containing 6% deionized formaldehyde were prepared in 1× MOPS running buffer, pH 7 (40 mM MOPS, 10 mM NaOAc, 1 mM EDTA, pH 8.0). The RNA sample (5 μL) was mixed with 15 μL RNA loading buffer (8 M urea, 6% formaldehyde, 1× MOPS running buffer, 0.05% bromophenol blue) and loaded onto the gel. Electrophoresis was performed in MOPS running buffer with circulation at 100 V for approximately 10 min, followed by 125 V until the dye front had migrated approximately 9 cm. Capillary transfer of separated RNA onto Hybond N+ membranes was performed overnight in 2× SSC (300 mM NaCl; 30 mM trisodium citrate dihydrate; pH 7.0) using a 1% agarose gel in 2× SSC as the buffer reservoir. After tRNA was cross-linked to the membrane by irradiation with 0.12 J/cm^2^ of UV light in a UVC 500 UV crosslinker (Hoefer). Membranes were prehybridized in hybridization buffer (90 mM NaCl, 50 mM NaH_2_PO_4_, pH 7.7, 5 mM EDTA, 5× Denhardt’s solution, 0.5% [wt/vol] SDS, 100 mg/mL sheared, denatured salmon sperm DNA) at 42°C for at least 1 h before addition of radioactively labeled DNA oligonucleotide and hybridization at 42°C overnight. After hybridization, membranes were washed at least three times in wash buffer (2× SSC, 0.1% SDS) at RT for 1 to 2 h, and radiation was detected by phosphor imaging in an Amersham Typhoon. For reprobing, hybridized oligonucleotides were removed by washing in boiling stripping buffer (0.1× SSPE, 0.5% SDS) until no remaining radiation was detected. Oligonucleotide probes were 5′ end labeled with [^32^P] by incubation of 150 pmol of oligonucleotide (1.5 μL of a 100 μM stock) with 10 U T4 polynucleotide kinase (PNK; Thermo Scientific) and 60 μCi ɣ-[^32^P]-ATP (6,000 Ci/mmol; Perkin Elmer) in a total volume of 10 μL at 37°C for 1 h. After 1 h, additional 10 U of PNK was added and incubation continued for 1 h.

Normalization of tRNA signals was performed as described in reference 11. For each lane, the bands corresponding to the target tRNA and the spike-in tRNA^selC^ were quantified using ImageQuant TL 8.2 image analysis software (GE Healthcare). The obtained target tRNA values were corrected by subtraction of the signal originating from endogenous expression of the same tRNA in the spike-in cell sample. The corrected values were then normalized by dividing the corrected value by the value of tRNA^selC^ from the same lane and plotted relative to the average of three samples taken during balanced growth.

tRNA levels in radiolabeled RNA samples obtained by hot phenol extraction were determined in the same manner, with the following exceptions. Prior to hybridization with oligonucleotide probes, the [^14^C] signal was visualized by phosphor imaging. During exposure, blots hybridized with [^32^P]-labeled oligonucleotide probes were covered with several layers of plastic sheets to minimize exposure of the storage phosphor screen to radioactivity originating from [^14^C]-labeled RNA. For normalization, the signal intensity of the target tRNA band was divided by the [^14^C]-signal of the entire tRNA fraction of the same lane, which was shown to be stable by the radiolabeling approach described above. The statistical significance of differences between individual time points after starvation/treatment and the steady-state level was assessed where applicable (*n* ≥ 3) by a two-tailed Student’s *t* test assuming unequal variances. To do so, the mean and standard deviation of all available steady-state samples (routinely three steady-state samples per sample series) were used.

### Data availability.

All raw data that are not shown in the figures or the supplementary information file are available upon request from Sine Lo Svenningsen (sls@bio.ku.dk).
