# Supplementary material for: tRNAs Are Stable After All: Pitfalls in Quantification of tRNA from Starved Escherichia coli Cultures Exposed by Validation of RNA Purification Methods
Source: mBio. 2023 Jan 4;14(1):e02805-22. doi: 10.1128/mbio.02805-22 (PMC9973347; doi:10.1128/mbio.02805-22)
Supplement: FIG S1 [file mbio.02805-22-s0001.pdf]

SUPPLEMENTARY FIGURE S1

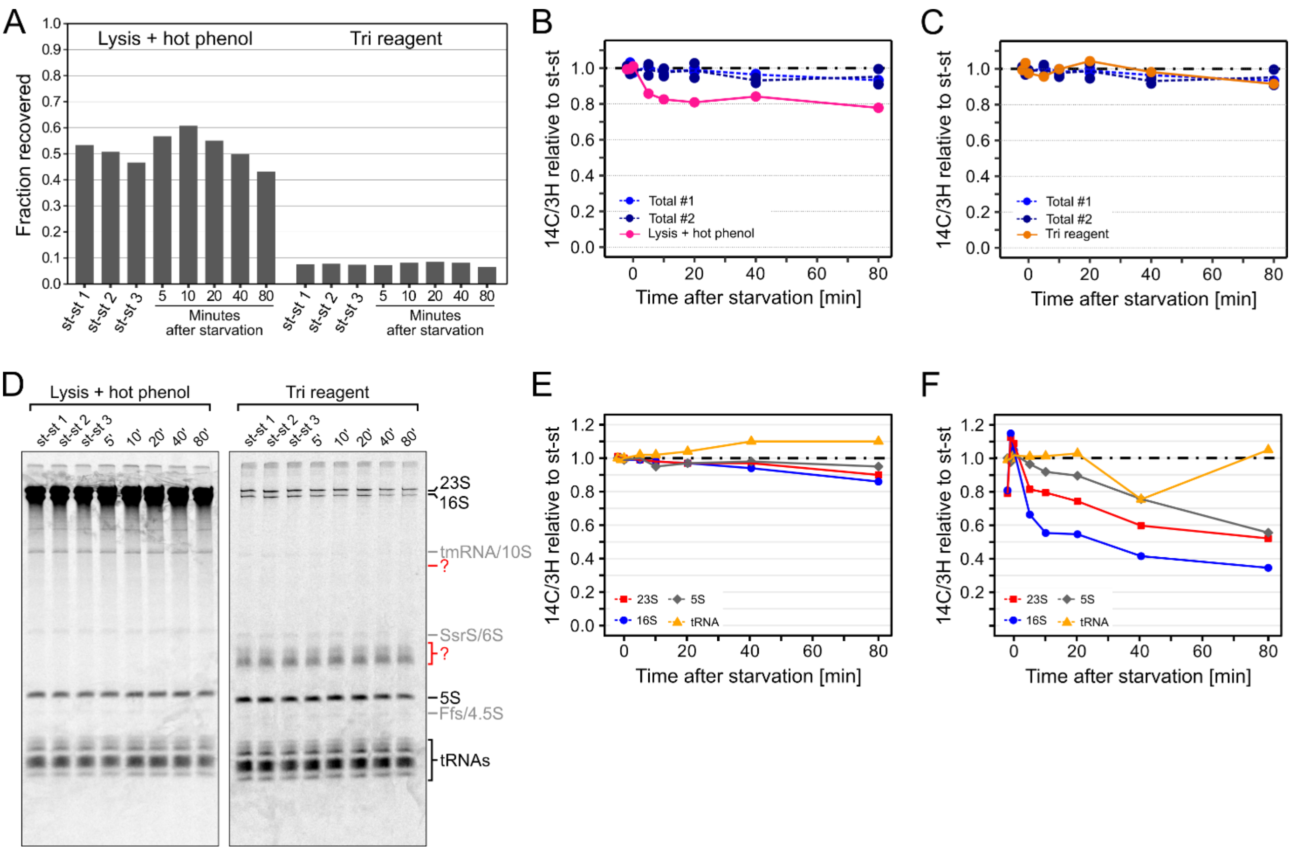

Supplementary Figure S1: Modification of hot phenol and TRI reagent extraction protocols does not affect extraction.

(**A**) Overall extraction efficiency of hot phenol extraction preceded by a lysis step (HP+), and the original TRI reagent protocol (TRI). Efficiency was determined as described in Figure 1 of the main manuscript. Shown RNA recovery in three samples obtained during steady-state growth (st-st 1-3) and 5, 10, 20, 40 and 80 min after isoleucine starvation.

(**B-C**) Efficiency of RNA extraction from starved cultures relative to steady-state cultures. Relative efficiency of HP+ (B) and the TRI (C) extractions was determined as the fraction of recovered RNA after starvation relative to the mean of three steady-state samples. Blue circles represent data points of two independent biological replicates of TCA-precipitable radioactivity, dotted lines represent the average of the two measurements. Pink and orange circles represent data points of one measurement. Dash-dotted line indicates the steady-state level.

(**D**) PAGE analysis of radiolabelled RNA obtained by HP+ and TRI extraction. ^{14}C-labelled RNA was separated on denaturing 5% polyacrylamide gels and detected by phosphor imaging. Bands corresponding to major RNA species (rRNAs and tRNAs) are indicated in black. Bands attributed to other abundant RNA species (tmRNA/10S RNA, 6S RNA and 4.5S RNA) are indicated in grey. Additional bands of unidentified origin visible in Tri reagent samples are marked with red questions marks.

(**E-F**) Efficiency of extraction of individual RNA species by HP+ (E) and TRI (F) extractions from starved cultures relative to steady-state cultures. RNA bands detected in (D) corresponding to 23S, 16S and 5S rRNA and tRNAs were cut out, RNase-treated and

incorporated radioactivity was measured. Shown is one biological replicate. Dash-dotted line indicates the steady-state level.
