# Supplementary material for: tRNAs Are Stable After All: Pitfalls in Quantification of tRNA from Starved Escherichia coli Cultures Exposed by Validation of RNA Purification Methods
Source: mBio. 2023 Jan 4;14(1):e02805-22. doi: 10.1128/mbio.02805-22 (PMC9973347; doi:10.1128/mbio.02805-22)
Supplement: FIG S2 [file mbio.02805-22-s0002.pdf]

SUPPLEMENTARY FIGURE S2

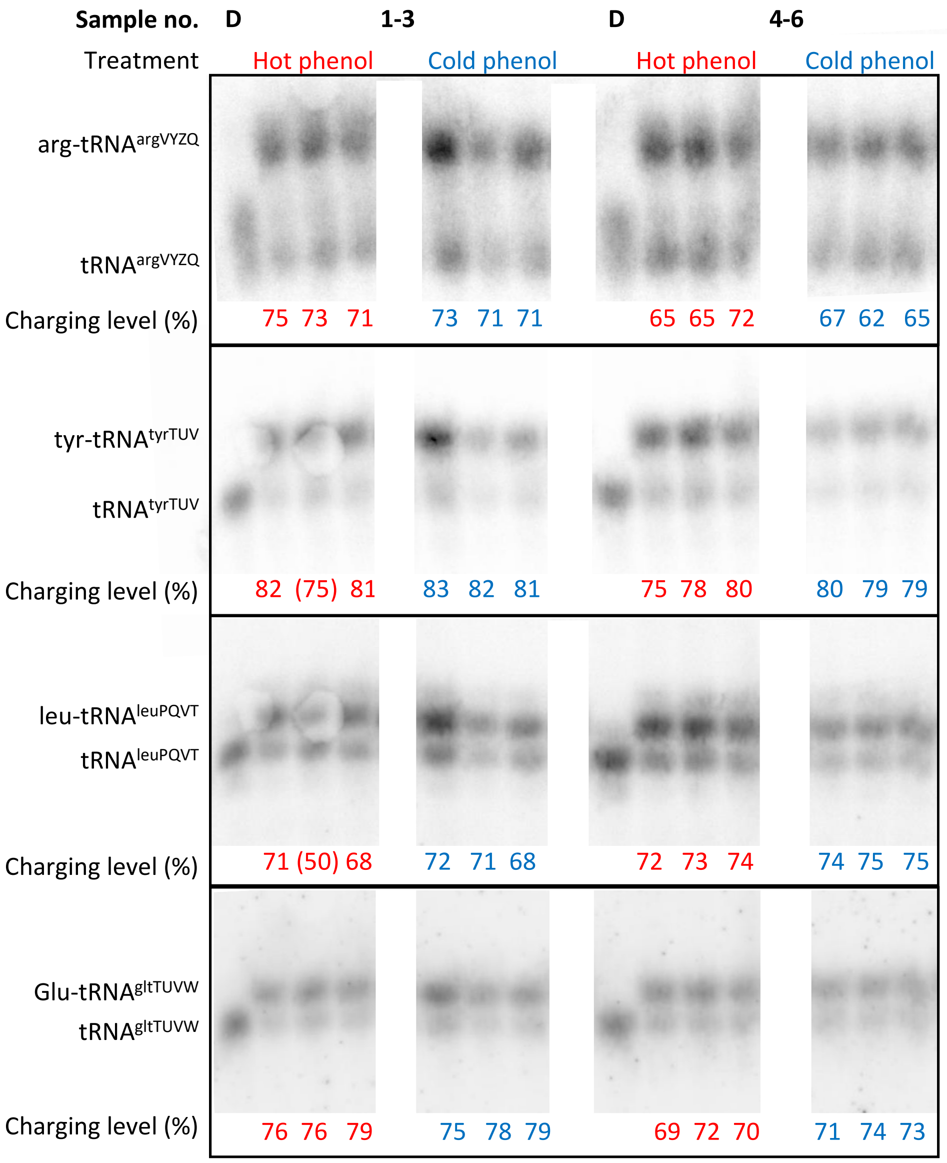

Supplementary Figure S2: Northern blots showing tRNA charging levels after extraction of RNA with hot or cold phenol. RNA was separated on a 50 cm sequencing gel and blotted as described (1, 2). Samples are the steady state samples from the two independent experiments #1 (sample 1-3) and # 2 (sample 4-6) from which data also are shown in **Figure 1**. Sample D is tRNA deacetylated with base (Tris·HCl pH 9.0; 1 h at 37°C). The membrane was probed with the probes: ArgVYZQ-anti, TyrTUV-anti, LeuPQVT and GltTUVW and the identities of the bands are indicated to the left of the blot. The charging levels are indicated under each lane. The charging levels were estimated by dividing the amount of radioactivity in the upper band with the total radioactivity in both bands. To estimate any difference in charging levels after extraction by the hot or cold phenol method the relative charging difference was calculated for each sample: $(Charging_{cold} - Charging_{hot})/Charging_{cold}$. The average of all differences was found to be = 0.004 and the $SD_{(n-1)}$ = 0.03. The two lanes where the values are in parentheses were omitted from the analysis due to RNA transfer irregularities that prohibited the correct estimation of the charging levels.
