# Supplementary material for: tRNAs Are Stable After All: Pitfalls in Quantification of tRNA from Starved Escherichia coli Cultures Exposed by Validation of RNA Purification Methods
Source: mBio. 2023 Jan 4;14(1):e02805-22. doi: 10.1128/mbio.02805-22 (PMC9973347; doi:10.1128/mbio.02805-22)
Supplement: FIG S3 [file mbio.02805-22-s0003.pdf]

SUPPLEMENTARY FIGURE S3

A

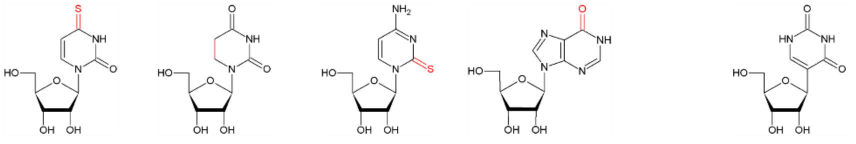

4-thiouridine (4) Dihydrouridine (D) 2-thiocytidine (%) Inosine (I) Pseudouridine (P)

tRNA^{argVYZQ} 5'-GCAUCCG4AGCUCAGCDGGADAGAGUACUCGG%UICG/ACCGAGCG7XCGGAGGTPCGAAUCCUCCCGGAUGCACCA-3'

ArgVYZQ-anti-alt

ArgVYZQ-5' ArgVYZQ-anti ArgVYZQ-3'

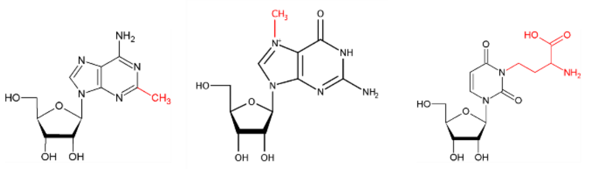

2-methyladenosine (/) 7-methylguanosine (7) 3-(3-amino-3-carboxypropyl)uridine (X)

B

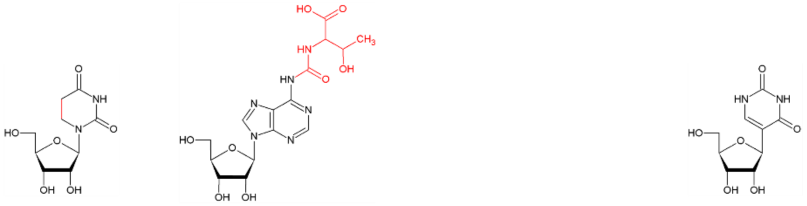

Dihydrouridine (D) N6-threonylcarbamoyladenosine (6) Pseudouridine (P)

tRNA^{ileTUV} 5'-AGGCUUGUAGCUCAGGDGGDDAGAGCGCACCCCUGAU6AGGGUGAG7XCGGUGGTPCAAGUCCACPCAGGCCUACCA-3'

IleTUV-anti-alt

IleTUV-5' IleTUV-anti IleTUV-3'

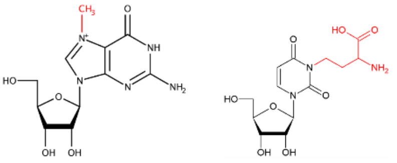

7-methylguanosine (7) 3-(3-amino-3-carboxypropyl)uridine (X)

C

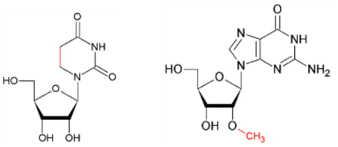

Dihydrouridine (D) 2′-O-methylguanosine (#)

tRNA^{leuPQVT} 5'-GCGAAGGUGGCGGAADD#GDAGACGCGCUAGCUUCAGKPGPUAGUGUCCUUACGGACGUGGGGGTPCAAGUCCCCCCCUCGCACCA -3'

LeuPQVT-5' LeuPQVT-anti LeuPQVT-3'

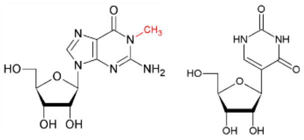

1-methylguanosine (K) Pseudouridine (P)

Supplementary Figure S3: RNA modification pattern of tRNAs that may influence probe binding.

(**A-C**) Sequence and modifications of tRNA^{argVYZ} (A), tRNA^{ileTUV} (note that the sequence shown is of tRNA^{ileTV}, which differs from tRNA^{ileU} at a single nucleotide) (B) and tRNA^{leuPQVT} (C) obtained from the MODOMICS database (3). Modified bases are in bold. Green font colour indicates modifications that do not affect Watson-Crick base pairing, red font colour indicates modifications that interfere with Watson-Crick base pairing, as inferred from position. Modifications present in the tRNA are depicted above and below the sequence, with adducts or changes in molecule structure indicated in red. Lines above or below the sequence indicate target regions for oligonucleotide probes used for northern blot hybridisation.
