# Supplementary material for: tRNAs Are Stable After All: Pitfalls in Quantification of tRNA from Starved Escherichia coli Cultures Exposed by Validation of RNA Purification Methods
Source: mBio. 2023 Jan 4;14(1):e02805-22. doi: 10.1128/mbio.02805-22 (PMC9973347; doi:10.1128/mbio.02805-22)
Supplement: TEXT S1 [file mbio.02805-22-s0010.pdf]

SUPPLEMENTARY METHODS

High resolution urea PAGE and Northern blotting for detection of tRNA fragments

RNA samples obtained from long-term starved MAS1081 was separated by denaturing polyacrylamide gel electrophoresis (PAGE) on 20% polyacrylamide gels buffered with 1 x TBE (100 mM Tris Base, 100 mM boric acid, 2 mM EDTA) and supplemented with 8 M urea. Samples were diluted in 2x loading buffer (95% formamide, 0.025% bromophenol blue, 0.025 xylene cyanol, 5 mM EDTA, 0.025% SDS). Electrophoresis was performed at 15 V/cm and RNA was transferred to Hybond N+ membranes by electroblotting in 1x TBE at 20 W constant. 5'-end radiolabelling, hybridization and detection of oligonucleotide probes was performed as described in the main article.

Starvation by depletion of nutrients

Starvation for carbon, phosphorus or nitrogen by depletion was induced by culturing cells in MOPS MM with a reduced concentration of glucose (0.07%), K_2HPO_4 (0.066 mM) or NH_4Cl (3 mM), respectively. The time point of starvation (t = 0 h) was estimated as the point of growth arrest as determined by measuring OD_{436}. In the case of phosphate depletion t = 0 h was defined as the point at which a marked decrease in growth rate was noticeable (i.e. exit from exponential growth), since the growth arrest was more gradual than during glucose and nitrogen starvation.

Supplementary Table S1: Strains used in the study

Strain	Description/Genotype	Source
MAS1081	*E. coli* K-12 MG1655 *rph*$^+$ *gatC*$^+$ *glpR*$^+$	(4)
MAS1190	MAS1081 *ΔargG ΔleuA ΔpyrE::tetR*	This work
MAS1074	*E. coli* BL21(DE3) pET11a(*selC*)	(1)
CF1651	*E. coli* K-12 MG1655 *ΔrelA251::kanR*	(5)
SAA21	MAS1190 *ΔrelA251::kanR*	This work

Supplementary Table S2: DNA oligonucleotides used in the study

Oligonucleotide	Sequence (5'->3')	Source
argVYZQ-3'	TGGTGCATCCGGGAGGATTCG	This work
argVYZQ-5'	TATCCAGCTGAGCTACGGATG	This work
argVYZQ-anti	TCCGACCGCTCGGTTCGTAGC	(2)
argVYZQ-anti-alt	GCTCGGTTCGTAGCCGAGTACTCT	This work
argW	CCTTAGGAGGGGCTCGTT	This work
asnTUVW	CAGTGACATACGGATTAACAGTC	This work
gltTUVW	CCTGTTACCGCCGTGAAAGGG	(2)
hisR	CACGACAACTGGAATCACAATCC	(2)
ileTUV-3'	TGGTAGGCCTGAGTGGACTTG	This work
ileTUV-5'	ACCACCTGAGCTACAAGCCT	This work
ileTUV-anti	ACCGACCTCACCCTTATCAG	(6)
ileTUV-anti-alt	TATCAGGGGTGCGCTCTAACC	This work
leuPQVT-3'	GTGCGAGGGGGGGGACTTGA	This work
leuPQVT-5'	CTACCAATTCCGCCACCTTCGC	This work
leuPQVT-anti	GTAAGGACACTAACACCTGAAGC	(2)
leuU	TATTGGGCACTACCACCTCAAGG	(2)
selC	ATTTGAAGTCCAGCCGCC	(1)
serT	CTTTCGGGTCGCCGGTTTTCA	This work
thrV	TGGGGACCTCACCCTTACCAA	(2)
trpT	CCCAACACCCGGTTTTGG	(6)
tyrTUV	TCGAACCTTCGAAGTCGATGA	(2)
valT	CTCCTTGTAAGGGAGGTGCTC	This work
valVW	CACCATGTCAAGGTGGTGCTC	This work
5S	ACACTACCATCGGCGCTAC	(7)
16S	GCTTTACGCCCAGTAATTCC	(7)
23S	TATCAGCCTGTTATCCCCGG	(7)

SUPPLEMENTARY REFERENCES

1. Stenum,T.S., Sørensen,M.A. and Svenningsen,S.L. (2017) Quantification of the Abundance and Charging Levels of Transfer RNAs in Escherichia coli. *JoVE J. Vis. Exp.*, 10.3791/56212.

2. Svenningsen,S.L., Kongstad,M., Stenum,T.S., Muñoz-Gómez,A.J. and Sørensen,M.A. (2017) Transfer RNA is highly unstable during early amino acid starvation in Escherichia coli. *Nucleic Acids Res.*, **45**, 793–804.

3. Boccaletto,P., Machnicka,M.A., Purta,E., Piątkowski,P., Bagiński,B., Wirecki,T.K., de Crécy-Lagard,V., Ross,R., Limbach,P.A., Kotter,A., *et al.* (2018) MODOMICS: a database of RNA modification pathways. 2017 update. *Nucleic Acids Res.*, **46**, D303–D307.

4. Gummesson,B., Shah,S.A., Borum,A.S., Fessler,M., Mitarai,N., Sørensen,M.A. and Svenningsen,S.L. (2020) Valine-Induced Isoleucine Starvation in Escherichia coli K-12 Studied by Spike-In Normalized RNA Sequencing. *Front. Genet.*, **11**.

5. Xiao,H., Kalman,M., Ikehara,K., Zemel,S., Glaser,G. and Cashel,M. (1991) Residual guanosine 3′,5′-bispyrophosphate synthetic activity of relA null mutants can be eliminated by spoT null mutations. *J. Biol. Chem.*, **266**, 5980–5990.

6. Dong,H., Nilsson,L. and Kurland,C.G. (1996) Co-variation of tRNA Abundance and Codon Usage inEscherichia coliat Different Growth Rates. *J. Mol. Biol.*, **260**, 649–663.

7. Fessler,M., Gummesson,B., Charbon,G., Svenningsen,S.L. and Sørensen,M.A. (2020) Short-term kinetics of rRNA degradation in *Escherichia coli* upon starvation for carbon, amino acid or phosphate. *Mol. Microbiol.*, **113**, 951–963.
