# Supplementary material for: tRNAs Are Stable After All: Pitfalls in Quantification of tRNA from Starved Escherichia coli Cultures Exposed by Validation of RNA Purification Methods
Source: mBio. 2023 Jan 4;14(1):e02805-22. doi: 10.1128/mbio.02805-22 (PMC9973347; doi:10.1128/mbio.02805-22)
Supplement: FIG S4 [file mbio.02805-22-s0004.pdf]

SUPPLEMENTARY FIGURE S4

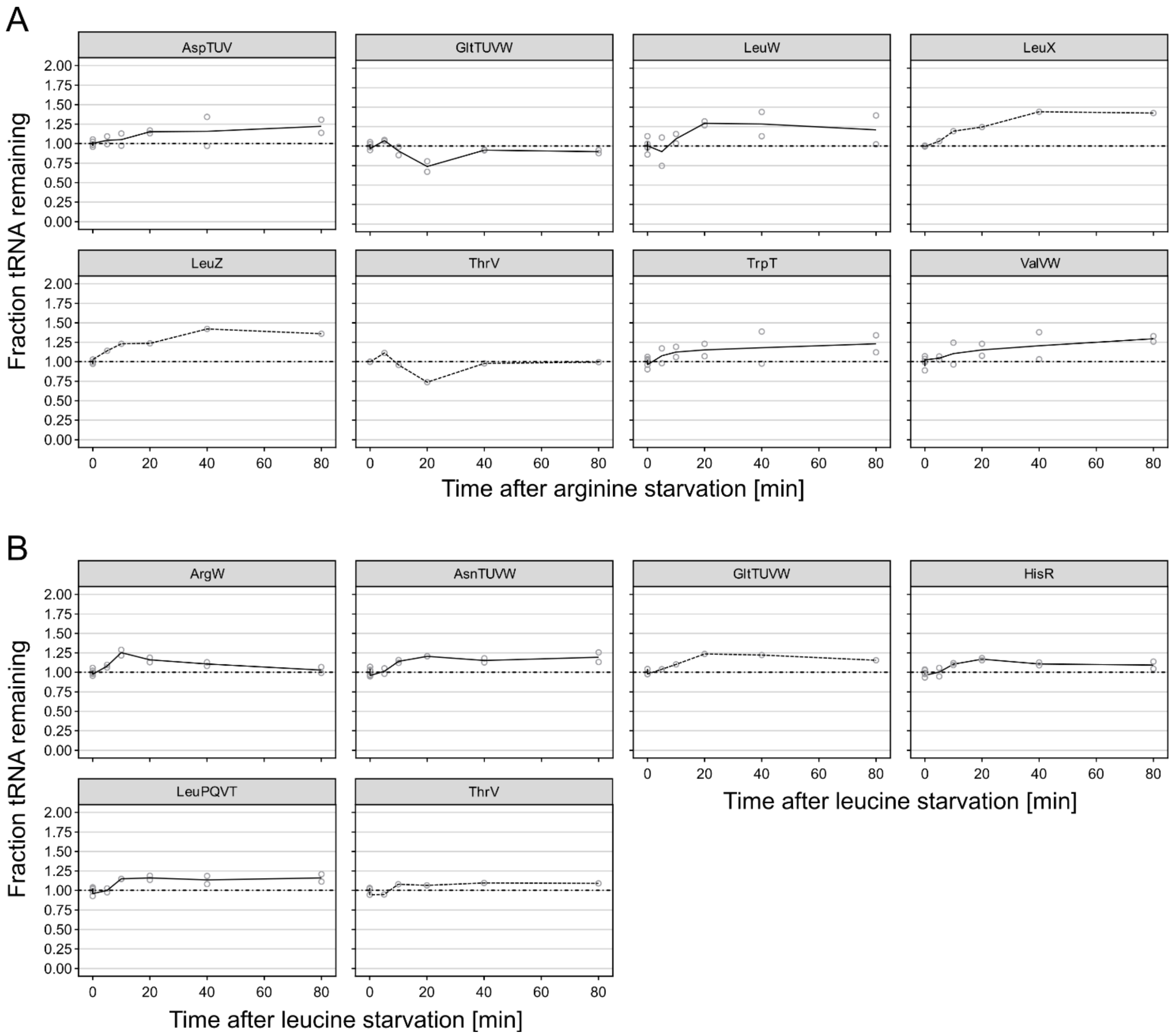

Supplementary Figure S4: tRNAs are stable during short-term arginine and leucine starvation. **A**) Quantification of additional tRNAs in MAS1190 after arginine starvation. tRNA levels were determined by Northern blot analysis as described for Figure 3. The fraction of tRNA remaining after starvation was calculated relative to three steady-state samples and normalized using a spike-in expressing large amounts of tRNA^{selC}. Circles represent data points of biological replicates, lines represent the mean of two biological replicates, dotted lines follow data points in case of one biological replicate. Dash-dotted line indicates the steady-state level.

B) Levels of selected tRNAs in MAS1190 after leucine starvation determined as described in (A).
