# Supplementary material for: tRNAs Are Stable After All: Pitfalls in Quantification of tRNA from Starved Escherichia coli Cultures Exposed by Validation of RNA Purification Methods
Source: mBio. 2023 Jan 4;14(1):e02805-22. doi: 10.1128/mbio.02805-22 (PMC9973347; doi:10.1128/mbio.02805-22)
Supplement: FIG S5 [file mbio.02805-22-s0005.pdf]

SUPPLEMENTARY FIGURE S5

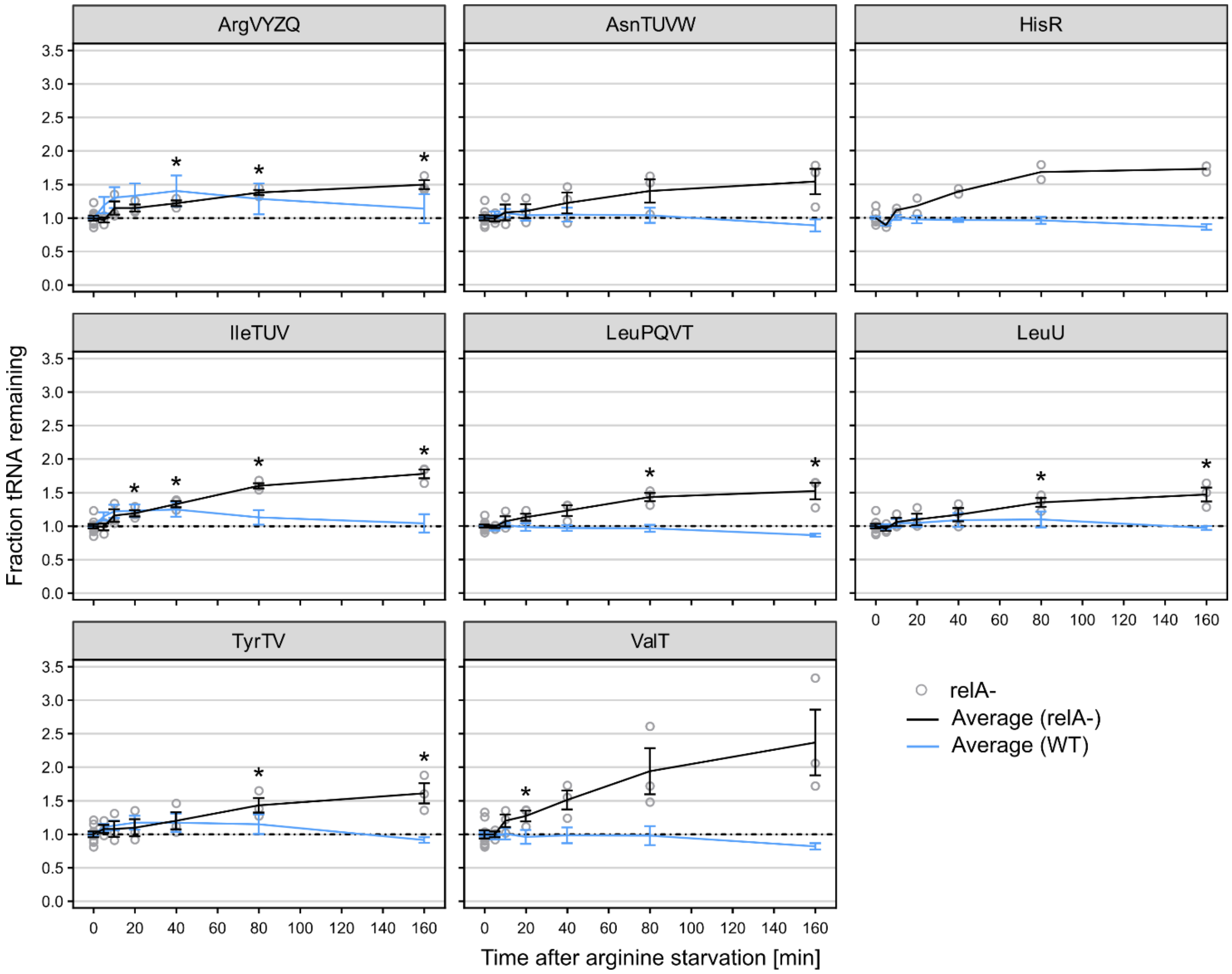

Supplementary Figure S5: tRNA levels increase after arginine starvation in a *relA*$^{-}$ strain.
Levels of selected tRNAs in SAA21 (*relA*$^{-}$) after arginine starvation were determined by Northern blot analysis. The fraction of tRNA remaining after starvation was calculated relative to three steady-state samples and normalized using a spike-in expressing large amounts of tRNA^{selC}. Lines represent the mean of three replicates except for HisR, where n = 2. Error bars indicate the standard error of the mean (SEM). Individual data points of replicates are shown as circles. *p-value < 0.05 as determined by a two-tailed student's t-test assuming unequal variances. The blue lines indicate the mean level of tRNAs in the *relA*$^{+}$ strain as presented in Figure 3 for comparison. Dash-dotted line indicates the steady-state level.
