# Supplementary material for: tRNAs Are Stable After All: Pitfalls in Quantification of tRNA from Starved Escherichia coli Cultures Exposed by Validation of RNA Purification Methods
Source: mBio. 2023 Jan 4;14(1):e02805-22. doi: 10.1128/mbio.02805-22 (PMC9973347; doi:10.1128/mbio.02805-22)
Supplement: FIG S6 [file mbio.02805-22-s0006.pdf]

SUPPLEMENTARY FIGURE S6

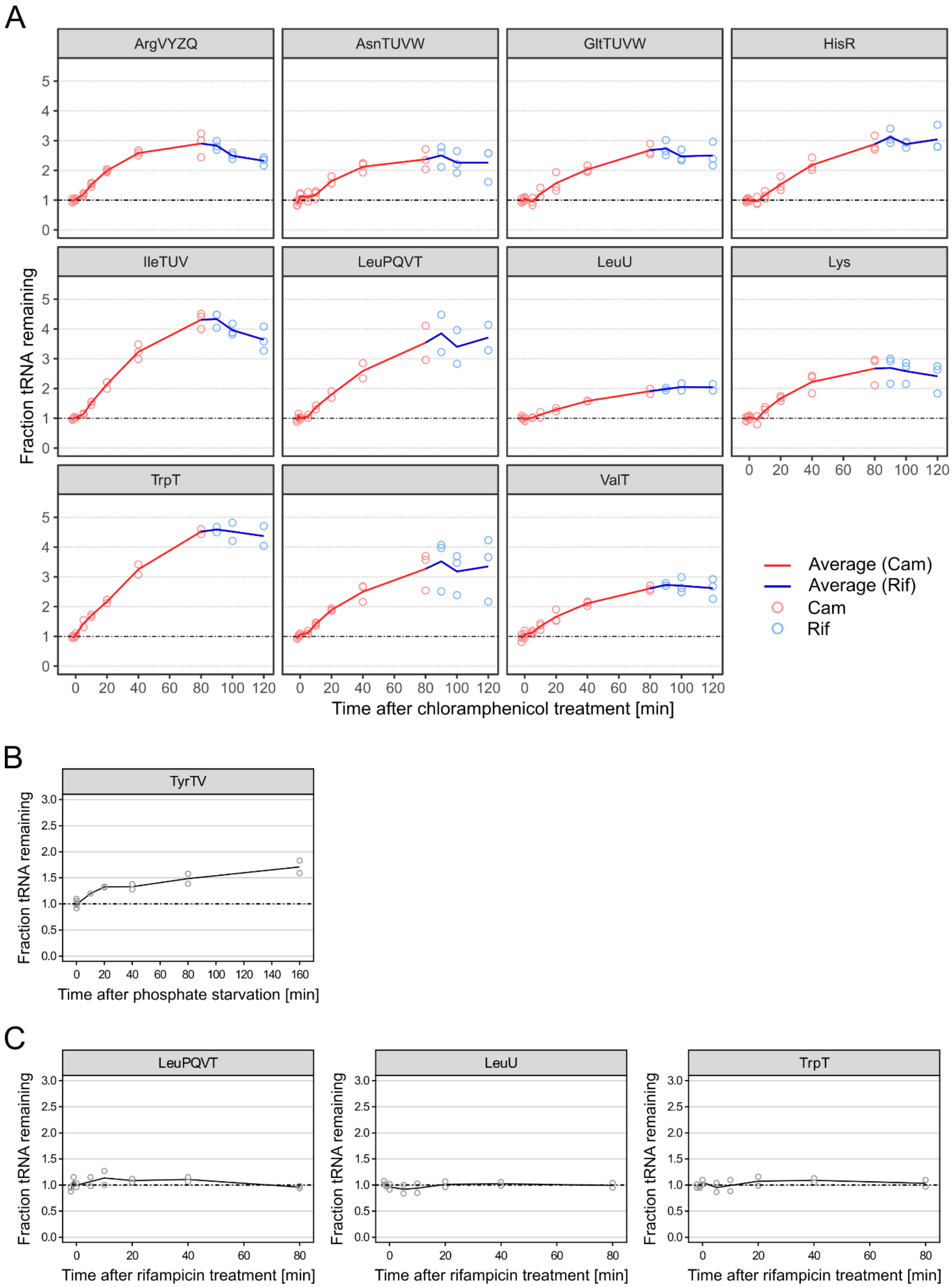

Supplementary Figure S6: tRNAs increase or are stable during chloramphenicol-induced repression of the stringent response, phosphate starvation and rifampicin treatment.

A) Levels of selected tRNAs after chloramphenicol treatment were determined by northern blot analysis. Chloramphenicol was added to steady-state cultures at time zero, followed by incubation for 80 min (red symbols). Then, rifampicin was added and incubation continued for 40 minutes (blue symbols). Note that the data shown for $\text{tRNA}^{argVYZQ}$ and tRNA^{ileTUV} was obtained with probes ArgVYZQ-anti and IleTUV-anti, respectively, thus likely underestimating the tRNA level after rifampicin treatment. Circles represent data points of biological replicates, lines represent the mean of the measurements. Dash-dotted line indicates the steady-state tRNA level.

B) Quantification of tRNA^{tyrTV} in MAS1081 after phosphate starvation as described in Figure 4. The fraction of tRNA remaining after starvation was calculated relative to three steady-state samples and normalized using a spike-in expressing large amounts of tRNA^{selC}. Circles represent data points of biological replicates, lines represent the mean of the measurements. Dash-dotted line indicates the steady-state level.

C) Levels of selected tRNAs in MAS1081 after rifampicin treatment determined as described in Figure 5.
