# Supplementary material for: tRNAs Are Stable After All: Pitfalls in Quantification of tRNA from Starved Escherichia coli Cultures Exposed by Validation of RNA Purification Methods
Source: mBio. 2023 Jan 4;14(1):e02805-22. doi: 10.1128/mbio.02805-22 (PMC9973347; doi:10.1128/mbio.02805-22)
Supplement: FIG S7 [file mbio.02805-22-s0007.pdf]

SUPPLEMENTARY FIGURE S7

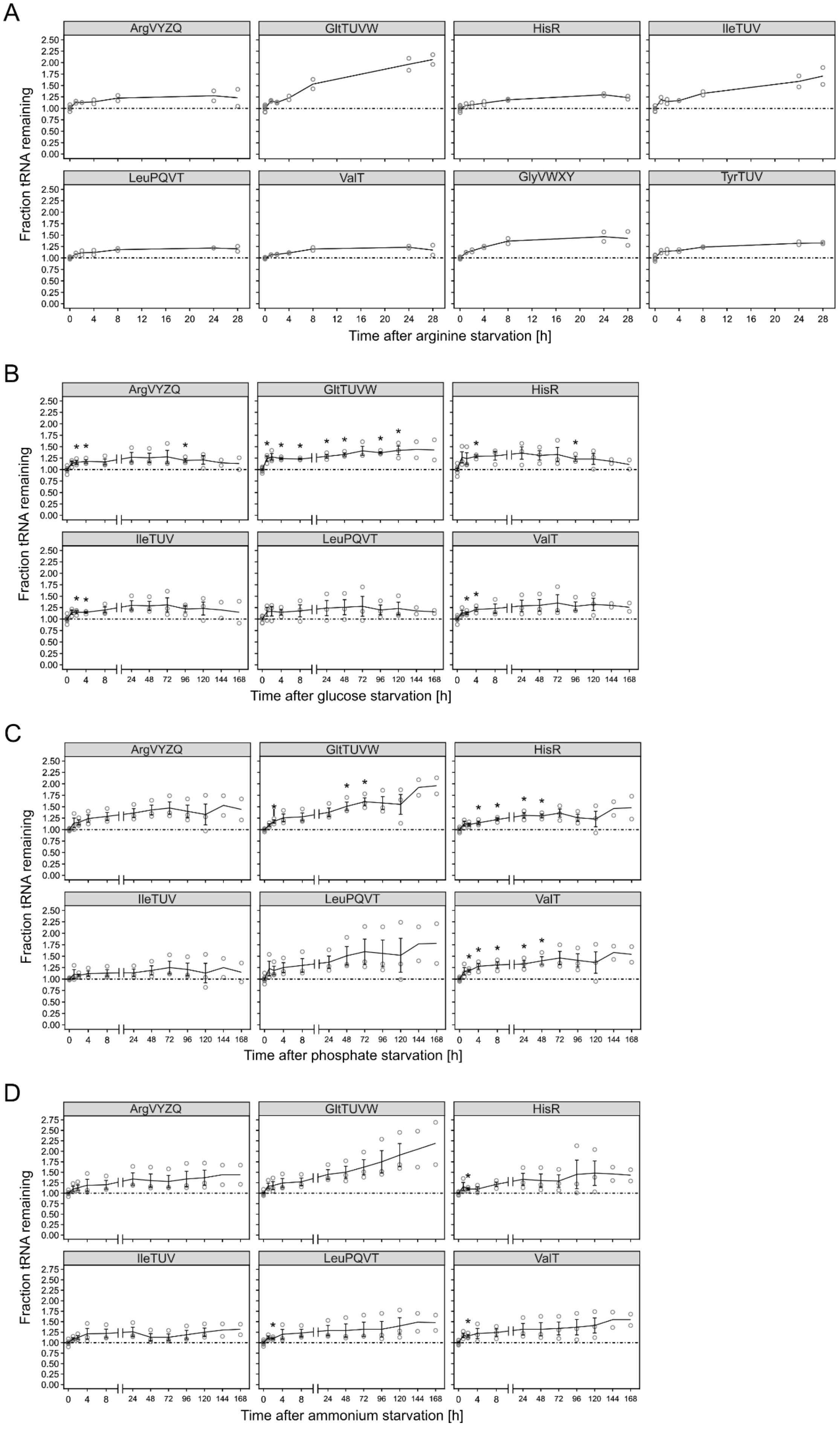

Supplementary Figure S7: Biological replicates of tRNA quantification after starvation for arginine, glucose, phosphate or ammonium

(**A**) Levels of selected tRNAs during long-term arginine starvation of MAS1190. Circles represent data points of two independent biological replicates; lines represent the mean of the measurements as presented in Figure 6A of the main article. Dash-dotted line indicates the steady-state level.

(**B**-**D**) Levels of selected tRNAs during long-term starvation for glucose (B), phosphate (C) and ammonium (D). Note that the x-axis is split into two segments for better resolution of time points in the first 8h of starvation. Lines represent the mean of three replicates except for t = 144-168h, where n = 2. Error bars indicate the standard error of the mean (SEM). Individual data points of replicates are shown as circles. *p-value < 0.05 as determined by a two-tailed student's t-test assuming unequal variances.
