# Supplementary material for: tRNAs Are Stable After All: Pitfalls in Quantification of tRNA from Starved Escherichia coli Cultures Exposed by Validation of RNA Purification Methods
Source: mBio. 2023 Jan 4;14(1):e02805-22. doi: 10.1128/mbio.02805-22 (PMC9973347; doi:10.1128/mbio.02805-22)
Supplement: FIG S8 [file mbio.02805-22-s0008.pdf]

SUPPLEMENTARY FIGURE S8

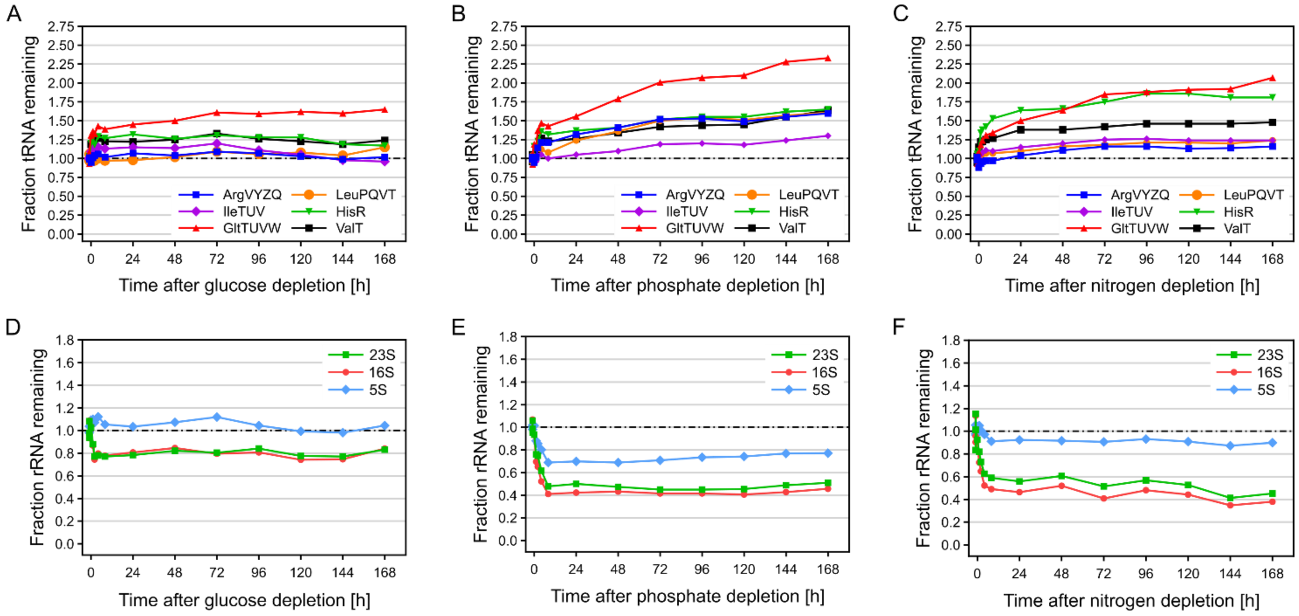

Supplementary Figure S8: tRNA and rRNA levels after starvation induced by depletion of glucose, phosphate or ammonium.

(**A-C**) Levels of selected tRNAs during long-term starvation for carbon (A), phosphorus (B) or nitrogen (C) induced by depletion. Cultures were grown in MOPS MM limited for glucose, phosphate or ammonium to induce starvation by depletion of the respective nutrient. Steady-state samples were taken during balanced growth (t = -1 h). t = 0 marks the onset of starvation estimated by the point of growth arrest, or a shift to a markedly lower growth rate. The addition of spike-in cells was calculated based on the optical density of the culture at t = 0 h. All other parameters of the experiment were identical to the data presented in Figure 6.

(**D-F**) As in (A-C), showing rRNA levels.
