# Supplementary material for: tRNAs Are Stable After All: Pitfalls in Quantification of tRNA from Starved Escherichia coli Cultures Exposed by Validation of RNA Purification Methods
Source: mBio. 2023 Jan 4;14(1):e02805-22. doi: 10.1128/mbio.02805-22 (PMC9973347; doi:10.1128/mbio.02805-22)
Supplement: FIG S9 [file mbio.02805-22-s0009.pdf]

SUPPLEMENTARY FIGURE S9

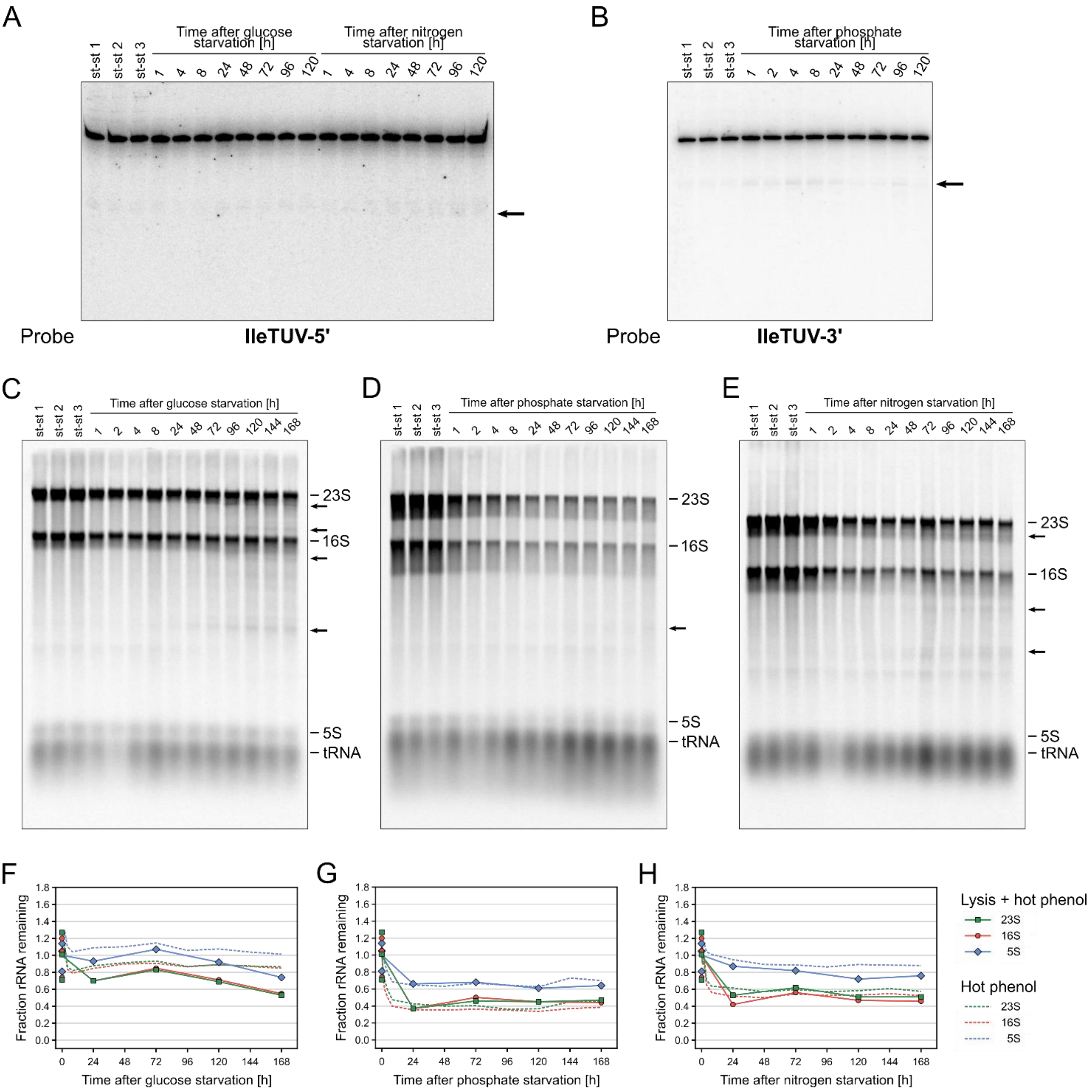

Supplementary Figure S9: Verification of tRNA and rRNA quantification during long-term starvation for glucose, phosphate or ammonium.

(**A-B**) High resolution northern blot analysis of RNA samples obtained from cultures starved for carbon, nitrogen or phosphate for five days. RNA was separated on 20% PAGE gels and subjected to northern blotting. An oligonucleotide probe targeting the 5'-end of tRNA^{IleTUV} was used to detect tRNA fragments in samples from carbon or nitrogen-starved cells (A), a probe targeting the 3'-end of tRNA^{IleTUV} was used to detect fragments in samples from phosphate-starved cells (B).

(**C-E**) Northern blots of radiolabelled RNA obtained from cultures starved for carbon (C), phosphorus (D) or nitrogen (E). ^{14}C-labelled RNA was separated on agarose gels, blotted onto nitrocellulose and detected by phosphoimaging. Bands corresponding to major RNA species (rRNAs and tRNAs) are indicated. Arrows indicate fragments likely originating from rRNAs.

(**F-H**) Levels of rRNAs during long-term starvation quantified from samples obtained by hot phenol extraction preceded by a lysis step. Levels were measured in cultures starved for carbon (F), phosphorus (G) or nitrogen (H). The legend for all three panels is indicated in panel (H). Lines with symbols indicate measurements obtained from the alternative protocol (lysis + hot phenol). Dotted lines indicate the mean levels as measured by the standard protocol using hot phenol extraction as presented in Figure 7.
